# Spatial and Temporal Resolution of the Oxygen-Independent Photoinduced DNA Interstrand Cross-Linking by a Nitroimidazole Derivative

**DOI:** 10.1021/acs.jcim.2c00460

**Published:** 2022-06-30

**Authors:** Abdelazim M. A. Abdelgawwad, Antonio Monari, Iñaki Tuñón, Antonio Francés-Monerris

**Affiliations:** † Departament de Química Física, Universitat de València, 46100 Burjassot, Spain; ‡ Université Paris Cité, CNRS, ITODYS, F-75006 Paris, France; § Université de Lorraine and CNRS, UMR 7019 LPCT, F-5400 Nancy, France

## Abstract

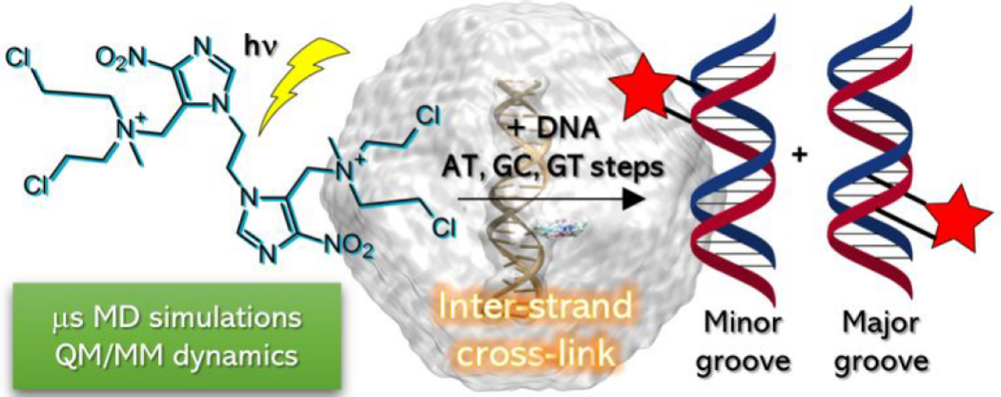

DNA damage is ubiquitous in nature and is at the basis of emergent treatments such as photodynamic therapy, which is based on the activation of highly oxidative reactive oxygen species by photosensitizing O_2_. However, hypoxia observed in solid tumors imposes the necessity to devise oxygen-independent modes of action able to induce DNA damage under a low oxygen concentration. The complexity of these DNA damage mechanisms in realistic environments grows exponentially when taking into account light absorption and subsequent excited-state population, photochemical and (photo)-redox reactions, the multiple species involved in different electronic states, noncovalent interactions, multiple reaction steps, and the large number of DNA reactive sites. This work tackles all the intricate reactivity of a photosensitizer based on a nitroimidazole derivative reacting toward DNA in solution under UV light exposition. This is performed through a combination of ground- and excited-state quantum chemistry, classical molecular dynamics, and hybrid QM/MM simulations to rationalize in detail the formation of DNA interstrand cross-links (ICLs) exerted by the noncanonical noncovalent photosensitizer. Unprecedented spatial and temporal resolution of these phenomena is achieved, revealing that the ICL is sequence-specific and that the fastest reactions take place at AT, GC, and GT steps involving either the opposite nucleobases or adjacent Watson–Crick base pairs. The N7 and O6 positions of guanine, the N7 and N3 sites of adenine, the N4 position of cytosine, and the O2 atom of thymine are deemed as the most nucleophile sites and are positively identified to participate in the ICL productions. This work provides a multiscale computational protocol to study DNA reactivity with noncovalent photosensitizers, and contributes to the understanding of therapies based on photoinduced DNA damage at molecular and electronic levels. In addition, we believe the depth understanding of these processes should assist the design of new photosensitizers considering their molecular size, electronic properties, and the observed regioselectivity toward nucleic acids.

## Introduction

DNA damage is a crucial phenomenon in biology and medicine and is naturally caused by several agents such as solar radiation or metabolic oxidative stress.^1−6^ Nevertheless, controlled DNA damage provides an opportunity for treatment. Photodynamic therapy (PDT) is a strategy in which cellular damage is selectively achieved through the application of light in combination with a photosensitizer,^7^ which is innocuous in the dark.^8−10^ Traditionally, the damage is exerted through the generation of singlet oxygen (^1^O_2_), a highly oxidative species formed by Type II photosensitization of triplet dioxygen.^11−14^ This feature becomes, however, an important limitation when dealing with cancerous tissues with marked hypoxia such as solid tumors, whose vascularization can be aberrant.^15−17^ Many types of photosensitizers with very diverse chemical structures have been developed and tested in the last years to overcome the hypoxia issue.^13,18−23^ In this context, the induction of biological damages must operate, at least partially, without the intervention of oxygen,^18,20^ leading to complex mixtures of chemical and photochemical events whose spatial and temporal disentanglement is challenging.

Intra- or interstrand DNA cross-linking induces oxygen-independent covalent links in DNA strands.^24,25^ In particular, DNA interstrand cross-links (ICLs) join the two antiparallel DNA strands. If unrepaired, ICLs block all biological processes that require DNA strand separation (such as DNA transcription and replication), ultimately inducing cell death.^24^ As a matter of fact, ICLs are at the basis of traditional chemotherapy such as in the case of cisplatin.^26,27^ Nevertheless, the lack of selectivity of chemotherapy induces strong secondary effects severely limiting the patients’ life quality and the drug acceptability. Controlling the formation of ICLs through its coupling with light irradiation adds the crucial selectivity needed to design anticancer therapies with milder side effects.^24^

The formation of intra- and inter-DNA cross-links have been tackled in the literature by using both static and dynamic computational methods. For instance, previous studies unravelled the reactivity that leads to the formation of inter- and intrastrand cross-links through a static quantum mechanics (QM) approach in model systems.^28−31^ Other works, explicitly accounting for the biological environments^32^ through classical molecular dynamics (MD)^33−37^ and quantum mechanics/molecular mechanics (QM/MM) methods,^38−40^ have assessed the impact of the cross-links on the DNA double strand helix dynamics. This is for instance relevant to find correlations between structural properties and difficulties in the cellular repair of the DNA lesions.^36^ Bignon et al. related the binding preferences of several polyamines with the propensity to form ICLs at specific DNA sites.^35^ On the contrary, studies dealing with reactivity in biological environments are much scarcer and are usually limited to the covalent linking between internal DNA nucleobases.^41^

The photoinduced ICL formation by a noncovalent photosensitizer requires the participation of several species in different electronic states, sometimes of different spin multiplicity, and involves the competition of multiple reaction channels. These facts, in conjunction with the intrinsic complexity of biological macromolecules, make the characterization of the mechanism a very intricate task that requires the combination of several theoretical methodologies. To the best of our knowledge, the spatial and temporal characterization of the sequence of events that take place from light absorption to the ultimate formation of the DNA ICLs has not been achieved until now. In the present work this is accomplished by employing a combination of *ab initio* and density functional theory (DFT)-based QM methods and classical and hybrid QM/MM MD simulations.

**Scheme 1:**
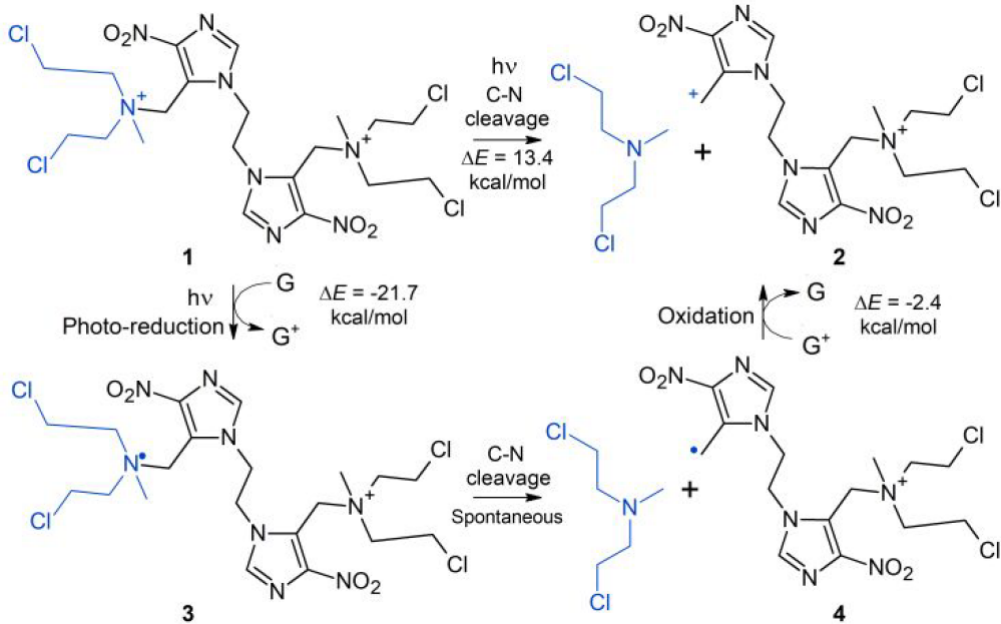
Chemical Structure of **1** and of the Different Derivatives G = guanine.

We have chosen a noncanonical photosensitizer based on a binitroimidazole backbone **1** (see Scheme 1) reported by Peng and co-workers.^42^ This selection is based on the following reasons:

- It is activated by light. Therefore, the systemic side effects associated with traditional chemotherapy are avoided.
- It has a dual action. Upon irradiation, **1** has a double mode of action: on the one hand, it generates free radicals able to react with DNA, and, on the other hand, it releases mechlorethamine (a nitrogenous mustard), which acts as a strong DNA alkylating agent.^42^ The observed lesions are DNA double strand breaks (DSB),^43^ i.e. the cleavage of the double strand, and DNA ICLs.^6,25,27,44^
- Oxygen is not involved in DNA damage.

Han et al.^42^ quantified the ICL yield to about ∼10% after the incubation of **1** and DNA for 2 h under irradiation at λ = 350 nm through nuclear magnetic resonance (NMR), high-resolution mass spectrometry, and liquid chromatography mass spectrometry. The photorelease of the nitrogenous mustard and the formation of reactive radicals was confirmed. Evidence for the occurrence of ICLs through the intervention of mechlorethamine was provided by analysis of the ICL stability and through experiments in the dark.^42^

This work characterizes, at an atomistic and electronic level, the dynamics of the DNA ICL formation mediated by free radicals and/or cations derived from **1**, i.e. **2** and **4** (Scheme 1). As shall be discussed in the following, this multiscale methodology allows the identification of the involved (excited) electronic states, the full characterization of the interaction between **1**, **2**, and **4** and DNA, and the satisfactory understanding of the regioselectivity (minor/major groove reactivity) and of the time scale of the ICL formation, providing a full and systematic assessment of the photoinduced DNA damage. Thus, the present work establishes a state-of-the-art computational approach to study photoinduced ICL formation exerted by noncovalent photosensitizers that accounts for the environmental effects and explicitly considers the kinetics of the processes, far beyond the static description of chromophore models. The current results offer insights into the process of ICL formation that could be extrapolated to other photosensitizers.^45−49^

## Results and Discussion

**Figure 1:**
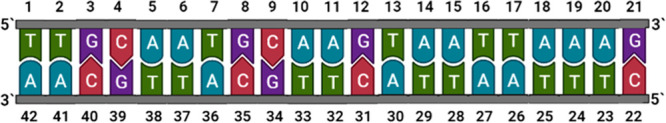
DNA sequence and nucleotide numbering.

**Scheme 2:**
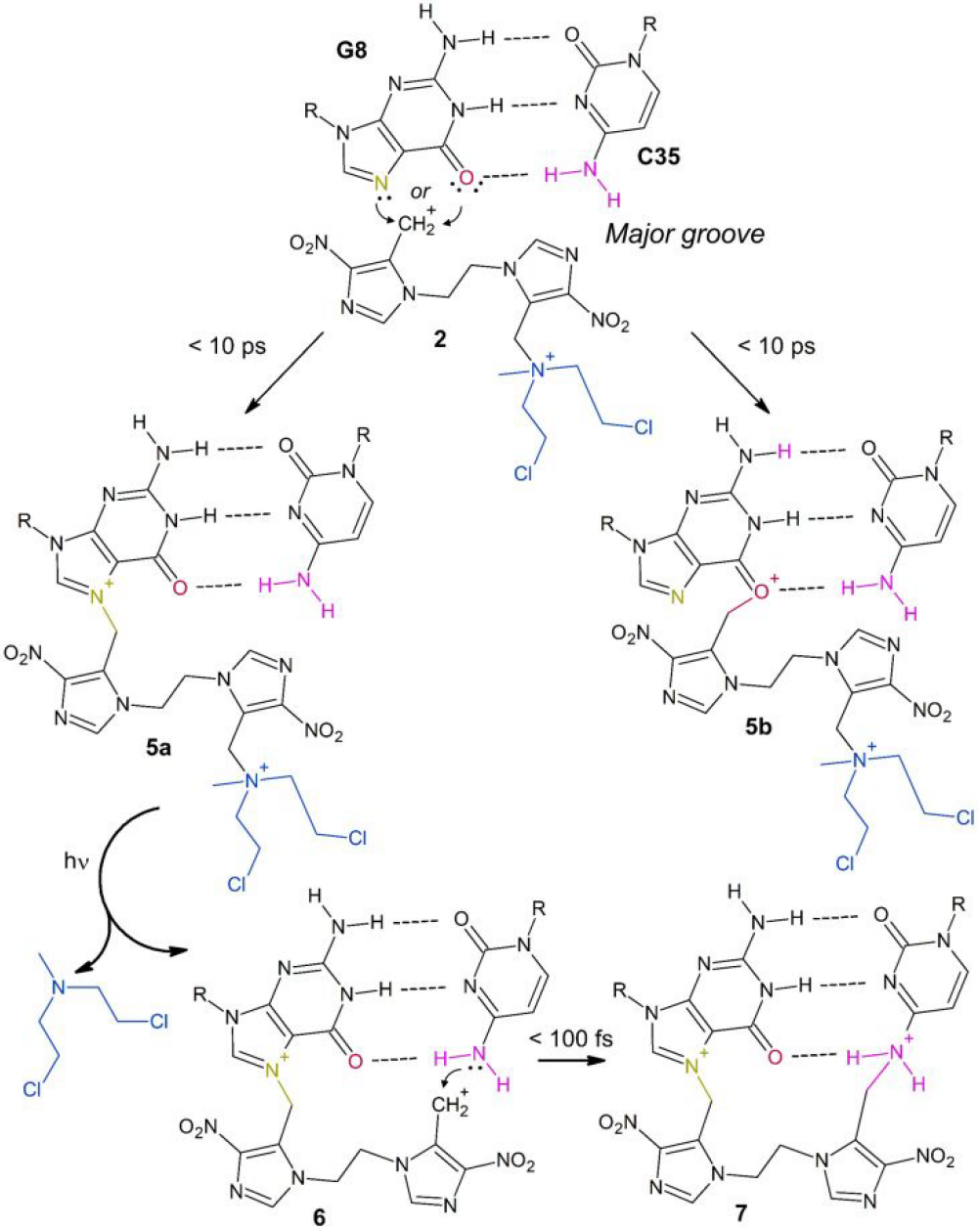
Mechanism for the Formation of the DNA ICL Product **7** between G8 and C35 R = DNA.

For the sake of readability, this section is organized in subsections according to the sequence of events that take place from the incubation of **1** with DNA to the formation of ICLs (Schemes 1 and 2). In all our simulations, we considered a model DNA oligomer with the sequence shown in Figure 1 that contains the central 21 steps of the DNA used in the experiments.^42^

### Optical Properties and C–N Cleavage Mechanism

The first DNA damage event entails the absorption of light followed by the (photo)release of mechlorethamine and the generation of the species **2** and **4**. These processes are studied by means of static time-dependent (TD)-DFT and complete-active-space second-order perturbation theory (CASPT2) calculations. Figure S2a shows the absorption spectrum of **1** determined with TD-B3LYP/6-31G*, a method validated with a CASPT2 benchmark (see the Computational Methodology section). The absorption maximum is located ca. 300 nm, in very good agreement with the experimental recording of 298 nm for neutral 4-nitroimidazole.^50^ At the experimentally irradiation wavelength (350 nm), the absorption is weak and is mainly caused by a small shoulder overlapping the main band tail. Analysis of the nature of the excited states at the Franck–Condon geometry (Figure S2b) reveal that the bright state is of π,π* character, as confirmed by CASPT2 (Tables S1–S3 and Figure S1). The low spin–orbit coupling values (<0.5 cm^–1^) confirm that the implication of triplet states in the photochemistry of **1** is negligible. A dark n_NO2_,π* state involving the transition from the oxygen lone pairs of the NO_2_ groups to the corresponding π* orbitals lies at ∼345 nm. Thus, the shoulder at ∼350 nm observed in the spectrum of Figure 1a will be mainly because of symmetry-forbidden n,π* absorptions. On the other hand, **4** shows two absorption bands. The lowest-energy one is significantly red-shifted as compared to the spectrum of **1**, possibly allowing its detection via nanosecond or microsecond time-resolved spectroscopy.

**Figure 2:**
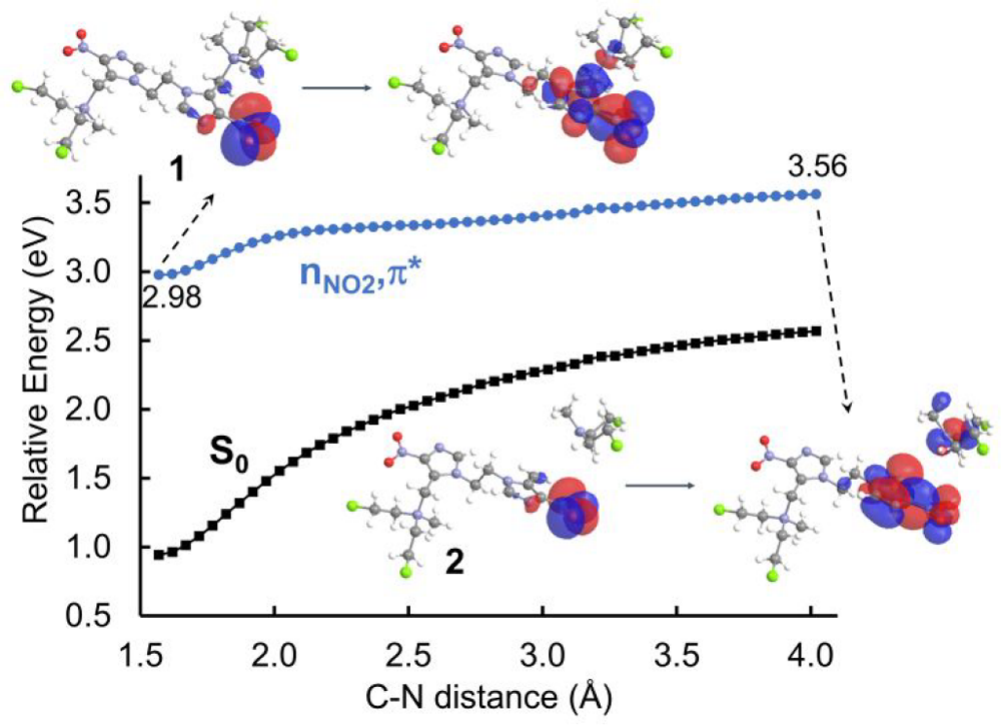
Relaxed scan of one C–N bond of **1** to yield **2** optimizing the S_1_ surface by using the TD-B3LYP/6-31G* method in implicit water solvation (PCM). The profile starts from the S_1_ equilibrium geometry. The NTO^51,52^ couples that represent the n_NO2_,π* excitations are shown at different C–N distances.

On the S_1_ surface, the cleavage of the C–N bond of **1** to release mechlorethamine and produce **2** involves an energetic penalty of ∼0.58 eV (∼13.4 kcal/mol), as shown in Figure 2. The energy barrier height is sufficiently small to justify the C–N cleavage in the excited state that takes place during the very long experimental irradiation times (2 h).^42^ The energy barrier for the mechlorethamine release on the S_1_ surface is smaller compared to the one on the ground state, justifying chemical stability in the dark. This different feature may be easily appreciated considering that the π* orbital localized on the nitroimidazole ring has antibonding character on the C–N bond (Figure 2).

An alternative bond breaking mechanism involves the one-electron reduction of **1** to yield **3**, an unstable species that spontaneously decomposes into mechlorethamine and **4** (Scheme 1). The C–N cleavage and mechlorethamine release in **3** is exothermic by −0.97 eV (−22.38 kcal/mol), as shown in the minimum energy path (MEP) displayed in Figure S3. Han et al.^42^ proposed that guanine, the DNA nucleobase with the lowest ionization potential (IP),^53^ could act as the electron donor. To justify this assumption, we may consider data collected in Table 1, which summarizes the thermodynamic parameters for some relevant one-electron redox and photoredox reactions. While the electron transfer from neutral guanine to **1** to produce **4** and the corresponding cationic guanine (G^+^) in the ground state is highly unfavorable (>50 kcal/mol), the photoreduction of 1* in its lowest S_1_ excited state is clearly exothermic (−21.74 kcal/mol). Even though no kinetic information is available about this process, it is reasonable to assume that this event will be operative in irradiated DNA + **1** mixtures, competing with the direct photocleavage of **1** shown in Figure 2. It is important to remark that the photoreduction of **1** is a bimolecular process and thus requires spatial proximity between 1* and a guanine, while the photorelease of **1** is a unimolecular process. Experimental observations detected **4** in the reaction medium in the presence of the (2,2,6,6-tetramethylpiperidin-1-yl)oxyl (TEMPO) radical quencher,^42^ indicating that the electron transfer between DNA and **1** actually takes place. After the photoreduction of **1** and the subsequent C–N scission (Figure S3), **4** can be oxidized to give rise to **2**. Two electron acceptors have been considered to mediate this process, namely neutral thymine (the DNA nucleobase with the highest electron affinity)^54,55^ and the positive cation of guanine, G^+^, formed in the previous reduction of 1*. Not surprisingly, G^+^ is the best electron acceptor, hence the self-repair of G^+^ is the most thermodynamically favorable channel leading to the carbocation **2**:
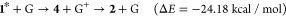


**Table 1:** Thermodynamics (Δ*E*) for Some Redox and Photoredox Reactions^a^

reaction	Δ*E* (kcal/mol)
**1** + G → **4** + G^+^	51.75
1* + G → **4** + G^+^	–21.74
**4** + T → **2** + T^–^	92.80
**4** + G^+^ → **2** + G	–2.44

a Asterisks denote that the species is considered in the lowest-lying singlet or doublet state (S_1_ for **1** and D_2_ for **4**), at its corresponding equilibrium geometry. The magnitudes have been computed with the M06-2X/6-31++G(d,p) method according to previous works on related systems.^56,57^

Thus, both the photorelease and the photoreduction/oxidation processes compete to produce **2**. While the former is a unimolecular process and fast enough to occur in the experimental time scale, the latter is bimolecular and, although thermodynamically favorable, requires spatial proximity between 1* and guanine.

### DNA-PS Noncovalent Interactions

The noncovalent interactions of **1**, **2**, and **4** (Scheme 1) with DNA were studied by using all-atom classical MD. DNA-photosentitizer close contacts are a prerequisite for the reactivity that induces DNA damage, and therefore they must be properly characterized. The B-DNA double strand model is a fragment of the longer oligonucleotide used in the experiments, specifically containing the central 21 steps (5′-TTGCAATGCAAGTAATTAAAG-3′) of the original sequence (Figure 1). The stability of the MD trajectories was evaluated by visual inspection and by analyzing the root-mean squared deviation (RMSD) for the DNA oligomer along the simulation (Figure S4), evidencing only limited and smooth fluctuations.

Several interaction modes have been identified in the multiple MD trajectories spanning ∼1 μs each, as summarized in Table 2. Note that the long time scales of these simulations are intended to maximize the sampling of the noncovalent interaction space and hence do not have any direct relation with the intermediate lifetimes. The single cations **1** and **4** show persistent interactions with the terminal A42–T1 and G21–C22 base pairs through efficient π-stacking between the two nitroimidazole and the nucleobase rings (Figures S5 and S6). This interaction is very favorable also because the molecular length of the photosensitizer matches the span of a DNA base pair. However, even though these interactions are expected to occur in model experiments with short oligonucleotides in solution,^42^ they are not biologically relevant because DNA terminal regions are scarce in cells. Given the proximity between the photosensitizer and the DNA, the persistence of this interaction may facilitate the photoreduction of 1* and the subsequent oxidation of **4** to produce **2** as shown in Table 1. On the other hand, the manual intercalation of **4** between two DNA base pairs led to the spontaneous release of the drug in few nanoseconds, hence confirming the lack of stability for this mode.

**Table 2:** Interaction Modes of Species **1**, **2**, and **4** and Their Preponderance over the Simulations

compound	run	interaction mode	approximated interaction time (ns)^a^
**1**	#1	none	140
		major groove	52
		minor groove	48
		terminal A42–T1	580
		terminal G21–C22	180
**2**	#1	none	64
		major groove	212
		minor groove	724
	#2	major groove	80
		minor groove	920
**4**	#1	none	52
		minor groove	6
		terminal G21–C22	920
	#2	none	52
		major groove	48
		minor groove	200
		terminal A42–T1	600
	#3	none	104
		major groove	36
		minor groove	20
		terminal G21–C22	660

a Refers to the approximated number of frames that show a given interaction type, not necessarily in a continuous time sequence. The total time per run is 1 μs.

**Figure 3:**
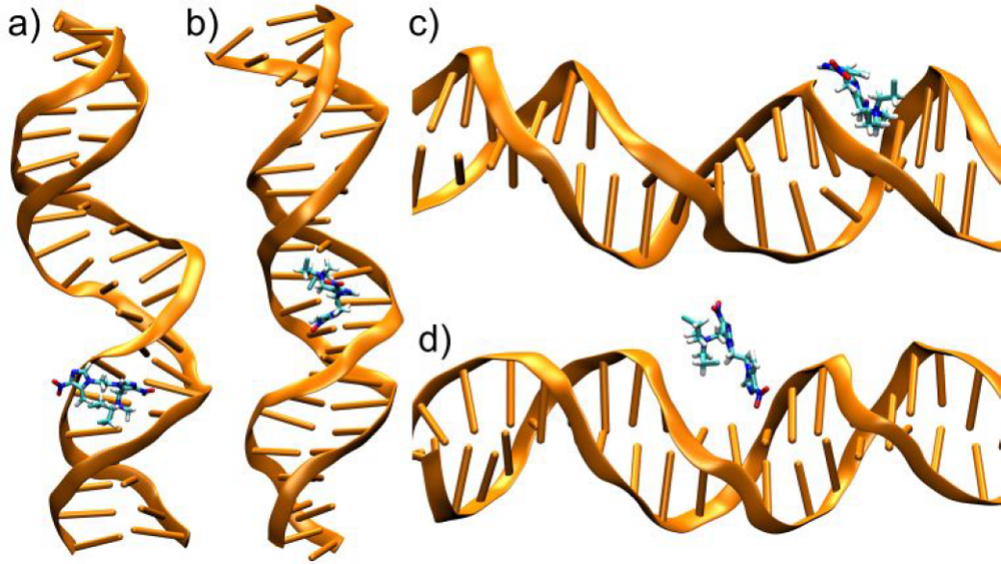
Representative snapshots of the interaction modes of **2** with minor (a and c panels) and major (b and d panels) grooves.

In stark contrast to **1** and **4**, **2** mostly interacts with DNA minor and, to a lesser extent, major grooves (Figure 3). The minor groove interaction can be very persistent. For instance, in run #2, the minor groove interaction, involving nucleotides A18 and A19, lasts for almost one microsecond (see Table 2). The different behavior as compared to **1** and **4** possibly correlates with an optimal distribution of the positive charges that maximizes the electrostatic interaction with the negatively charged phosphate backbone. Bignon et al. found a clear relationship between the protonation state (and hence positive charges) of several polyamines and their corresponding interaction modes with DNA.^35^ Note that **1** and **4** also exhibit minor and major groove interaction modes (Table 2) albeit with a persistence of a few tens of nanoseconds, because this mode of action is not dominant.

The noncovalent interaction energies between **1** and the DNA nucleotides (Figure S7), at the nucleotide level, correlate very well with the distances between the centers of mass of **1** and the nucleotides, i.e. the closer the distance, the stronger the interaction energy (Figure S8). These data quantify the aforementioned interaction behavior for each species. The corresponding distribution of distances evidence that **1** mostly interacts with the terminal A42–T1 base pair, while **4** mostly stays close to the terminal G21–C22 pair (Figure S10). Product **2** interacts mostly with nucleotides #10–12, #26–28 and, more persistently, with #18 and #19, as mentioned (Figure S9). The noncovalent interactions include electrostatic interactions but also hydrogen bonding, as evidenced in Figure S11.

In the case of **2**, no clear preference for any nucleobase has been found, indicating that the interactions are rather sequence independent, as expected for groove-driven structures.

### Intrinsic Reactivity with DNA Nucleobases

**Chart 1:**
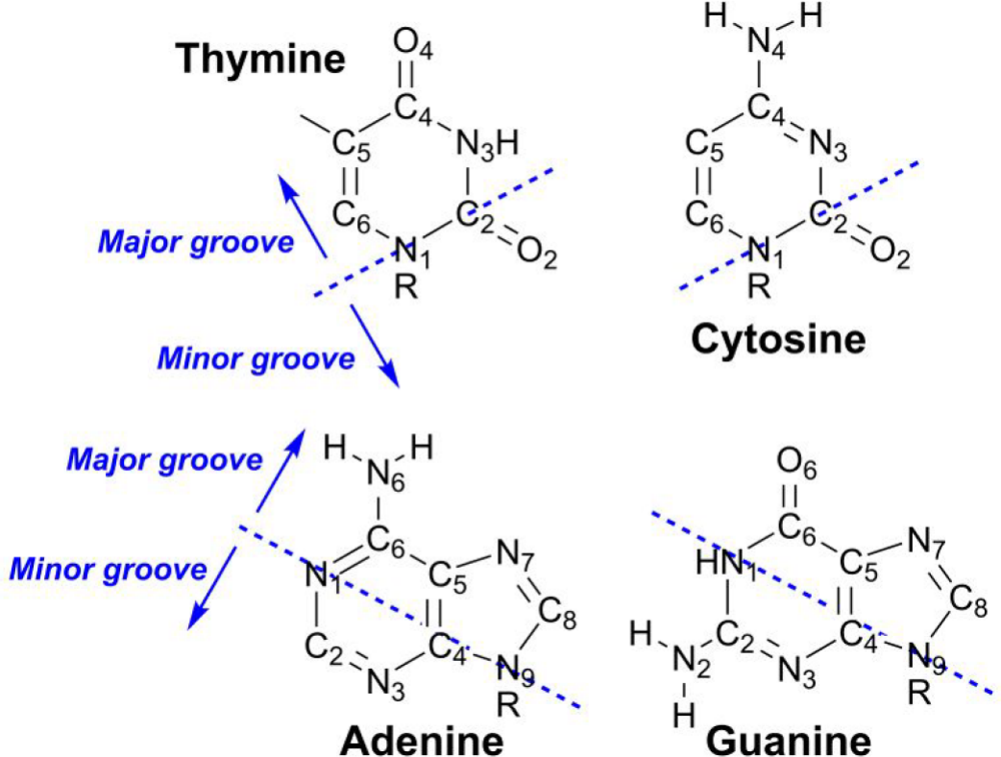
Atom Numbering of the Four DNA Nucleobasesa

The dynamics of the reaction between the species **2** and **4** and DNA will be mostly driven by the shape of the potential energy surfaces (PESs) associated with the formation of the covalent bonds at the different atom positions (Chart 1), given the sequence-independent modes of DNA interaction.

a The molecular regions exposed either toward the minor or major DNA grooves are also shown.

Before explicitly treating the role of the environment, we have modeled the addition of **4** to the C5 and C6 positions of thymine to (i) choose a suitable QM model size for the photosensitizer and (ii) to validate an appropriate DFT method to describe the reactions. The benchmark analysis of the size of the QM partition (Figure S12 and Table S4) and of different DFT functionals (Table S5) can be found in the Supporting Information. The M06-2X functional provided the best description of the energy barriers and therefore will be subsequently used to model the reactivity of **2** and **4** with the four DNA nucleobases, in agreement with previous works on related reactions such as the OH radical addition to DNA nucleobases.^58−61^

Tables 3 and S7 list the thermodynamics (Δ*G*) and kinetics (Δ*G*^‡^) for the reactions of **2** and **4**, respectively, with all DNA nucleobases. Whereas most of the reactions of **2** are highly exergonic and barrierless or almost barrierless (Table 3 and Figure S13), the reactivity of **4** is clearly slower and thermodynamically less favorable (Table S7). Greenberg and co-workers found similar results for the radical/carbocation localized in thymine.^62^

**Table 3:** Gibbs Energy Difference (kcal/mol) between Reactants and Products (Δ*G*) for the Addition Reaction of **2** to the Most Relevant Positions of the Four DNA Nucleobases^a^

reaction channel	Δ*G*
thymine	
O2	–15.20
cytosine	
O2	–30.61
N3	–37.51
guanine	
N3	–25.77
O6	–24.50
N7	–39.43
adenine	
N1	–36.38
N3	–36.47
N6	–23.05
N7	–30.24

a This table is a summary of Table S6.

The most exergonic routes are the additions of **2** to adenine (N1, N3, and N7 positions), guanine (N3, O6, and N7 positions), and cytosine (N3 and O2 positions). In general, the carbon sites are less favorable, while the reactivity tendency of thymine is, globally, less pronounced. From these static, and implicit solvent-based results, as well as the absence of a pronounced selectivity for the formation of the noncovalent aggregates between **2** and DNA (see previous subsection), the most exergonic routes should drive the regioselectivity of the reaction of **2** toward DNA in solution. This hypothesis is tested in the next subsections, in conjunction with the influence of the temperature and the anisotropic set of forces present in the complex environment, which must be taken explicitly into account.

### Reactivity in Biological Media: Covalent Linking to DNA

The kinetics of the reaction of **2** with DNA will be influenced not only by the shape of the electronic PESs but also by environmental effects such as the accessibility to the nucleobase positions, the electrostatic anisotropy, solvation/desolvation processes, and the collective influence of the temperature and the surrounding forces. To account for all these factors, the dynamics of the reaction of **2** with DNA nucleobases has been studied by means of hybrid QM/MM simulations, in which the QM partition spans the small model shown in Figure S12, i.e. the nitroimidazole ring of **2** and the DNA nucleobase. The reactivity of each nucleobase position is quantified by statistical analyses of multiple QM/MM trajectories.

**Figure 4:**
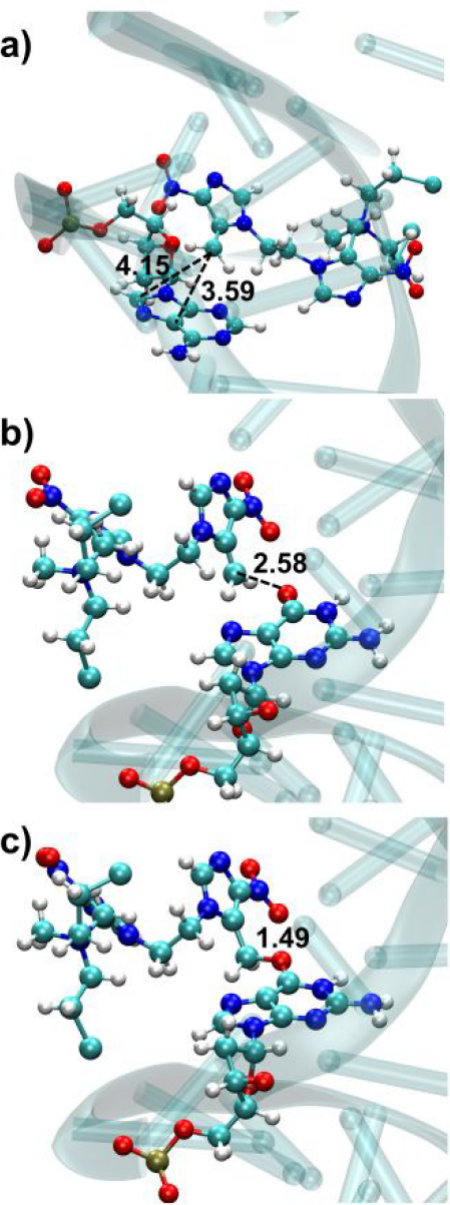
Representative snapshots of the reactivity of **2** toward DNA. (a) Initial frame (*t* = 0 ps) of one of the simulations of **2** close to A18. C^+^–C5 and C^+^–C8 distances are shown. No reaction was observed in 10 ps. (b) Initial frame (*t* = 0 ps) of one of the simulations of **2** close to G8. C^+^–O6 distance is shown. (c) Snapshot at *t* = 0.25 ps for one of the simulations of **2** close to G8. C^+^–O6 distance is shown. All distances are in Å.

Initial structures and atom velocities were extracted from major and minor groove interactions taking place in the classical MD simulations (summarized in Table 2) with relatively short distances between **2** and the target nucleobases (Figure 4). Each QM/MM run was propagated for 10 ps except in the case of earlier reaction. In that case, the simulation was stopped after the irreversible formation of the covalent bond.

Table 4 summarizes the results of the QM/MM simulations, which are much more detailed in Tables S8–S11. The carbocation **2** reacts with both major and minor DNA grooves. The major groove reactivity is dominated by the N7 and O6 positions of guanine (12 reactions out of 16 runs, combining the runs on both G8 and G34 nucleobases), yielding the intermediate products **5a** and **5b**, respectively (Scheme 2). Both reactions are competitive, even though considering the larger number of reactive trajectories and the shorter average reaction times, it seems that the reaction with O6 may be more favorable than with N7 (Table 4). The N4 position of cytosine (a primary amine) is also sufficiently nucleophilic to react with the carbocation of **2** within our simulation time scale, although to a lesser extent with respect to the guanine sites (only three reactions out of 15 simulations).

**Table 4:** Number of Reactions and Average Time until the Reaction Occurs Between 2 and DNA as Obtained with QM/MM Dynamics^a^

nucleobase	groove	position	observed reactions/total number of runs	average reaction time (ps)
Gua 8	major	N7	1/7	9.25
		O6	5/7	0.74
Gua 34	major	N7	3/9	4.77
		O6	3/9	0.92
Cyt 9	major	N4	2/8	5.32
Cyt 35	major	N4	1/7	0.25
Ade 6	major	N7	0/3	NR
		N6	0/3	NR
Ade 14	major	N7	0/1	NR
		N6	0/1	NR
Thy 13	major	C5	0/9	NR
		O4	1/9	0.85
Thy 25	major	C5	0/7	NR
		O4	0/7	NR
observed major groove reactions: 16/51				
Thy 25	minor	O2	3/7	3.77
Gua 34	minor	N2	1/8	7.95
Cyt 35	minor	O2	1/7	9.15
Ade 10	minor	N3	5/8	3.14
Ade 18	minor	N3	5/8	7.29
observed minor groove reactions: 15/54				

a NR stands for no reaction observed in the 10 ps simulation. *t* = 0 corresponds to the first frame of the run. This is a summary of Tables S8–S11. Note that the N7 and O6 positions of guanine and the C5 and O4 positions of thymine are, spatially, very close each other. Therefore, a single QM/MM run can show reaction with one or another site.

On the other hand, the positions of thymine facing the major groove (C5 and O4) are clearly the less reactive in biological media. Interestingly, these sites are also the less exothermic paths in the QM model (Table S6), suggesting a probable correlation between these two factors. The major groove reactivity on the N7 position of adenine is expected to be similar to that of the N7 site of guanine. Table 4 shows no reactivity on the former most likely because of the low number of QM/MM runs. The statistics could not be enlarged because only a few close contacts between the reactive carbocation of **2** and the N7 atom of adenine were found in the classical MD runs.

**Scheme 3:**
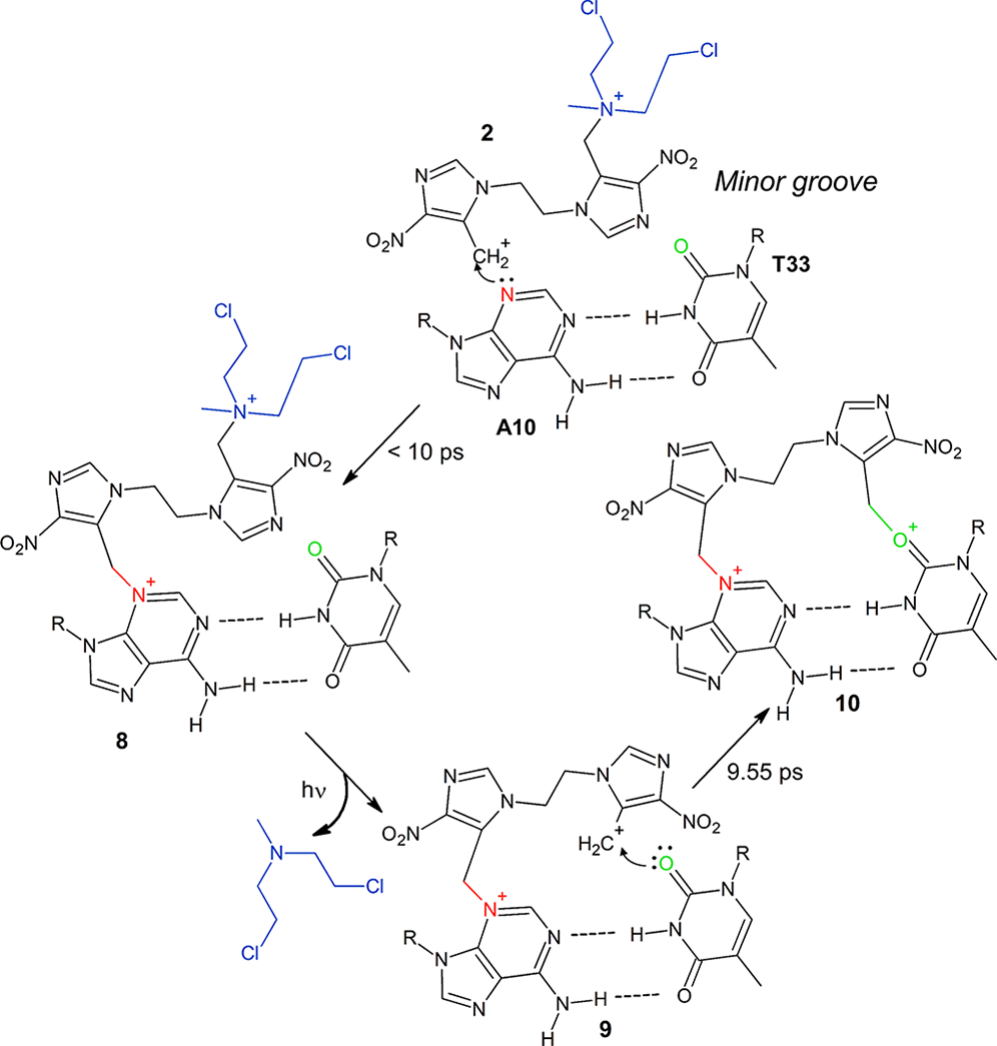
Formation of the DNA ICL Product **12** between A10 and T33 R = DNA.

The minor groove reactivity is dominated by the N3 position of adenine, which shows the largest number of reactive events (Table 4) to give the intermediate product **8** (Scheme 3). The high exergonicity of this channel (Table 3) reinforces the relationship encountered between exergonicity in the QM system and QM/MM reactivity. This correlation is most likely a consequence of the steeper electronic PES derived from the much larger energy difference between product and reactants.

In the minor groove, the N2 and O2 positions of guanine and cytosine, respectively, are much less reactive (Table 4). Only one reaction out of 7 or 8 simulations is observed at each nucleobase. On the other hand, only a very few contacts between the reactive carbocation of **2** and the N3 position of guanine were found (none of them below 4 Å). This fact, in combination with the less exergonicity of this channel as compared to the N3 position of adenine (Table 3), suggests that this site is not as relevant as the N3 position of adenine. Therefore, it can be safely concluded that the major groove reactivity takes place, in preference order, at guanine, adenine, and cytosine, whereas the minor groove reactions mostly occur at AT base pairs.

Data listed in Table 4 shows the occurrence of the DNA lesions **5a**, **5b**, and **8**, among others (Schemes 2 and 3). These are stable intermediates in the formation of ICLs. It is therefore expected that the remaining mechlorethamine unit is released from these species, leading to reactive species analogous to **2** and **4** (Scheme 1) even though this time covalently linked to DNA (species **6** and **9**). The molecular mechanism is considered very similar to the C–N photodissociation shown in Figure 2 or to the photoreduction/oxidation reactions shown in Table 1. However, given the covalent link between the nitroimidazole derivative and DNA, assuring proximity with the DNA nucleobase, the one-electron redox reactions are expected to be faster with respect to the free photosensitizer **1**.

### Interstrand Cross-Link Formation

In the case of **5a**, a major groove lesion, the product of the second mechlorethamine release is **6** (Scheme 2), a carbocation analogous to **2**, which is expected to react with a nearby guanine or cytosine to form the final ICL product. This reactivity has been studied by means of additional classical MD and QM/MM simulations, also with the aim to determine the most accessible reaction hotspots in the surroundings.

**Figure 5:**
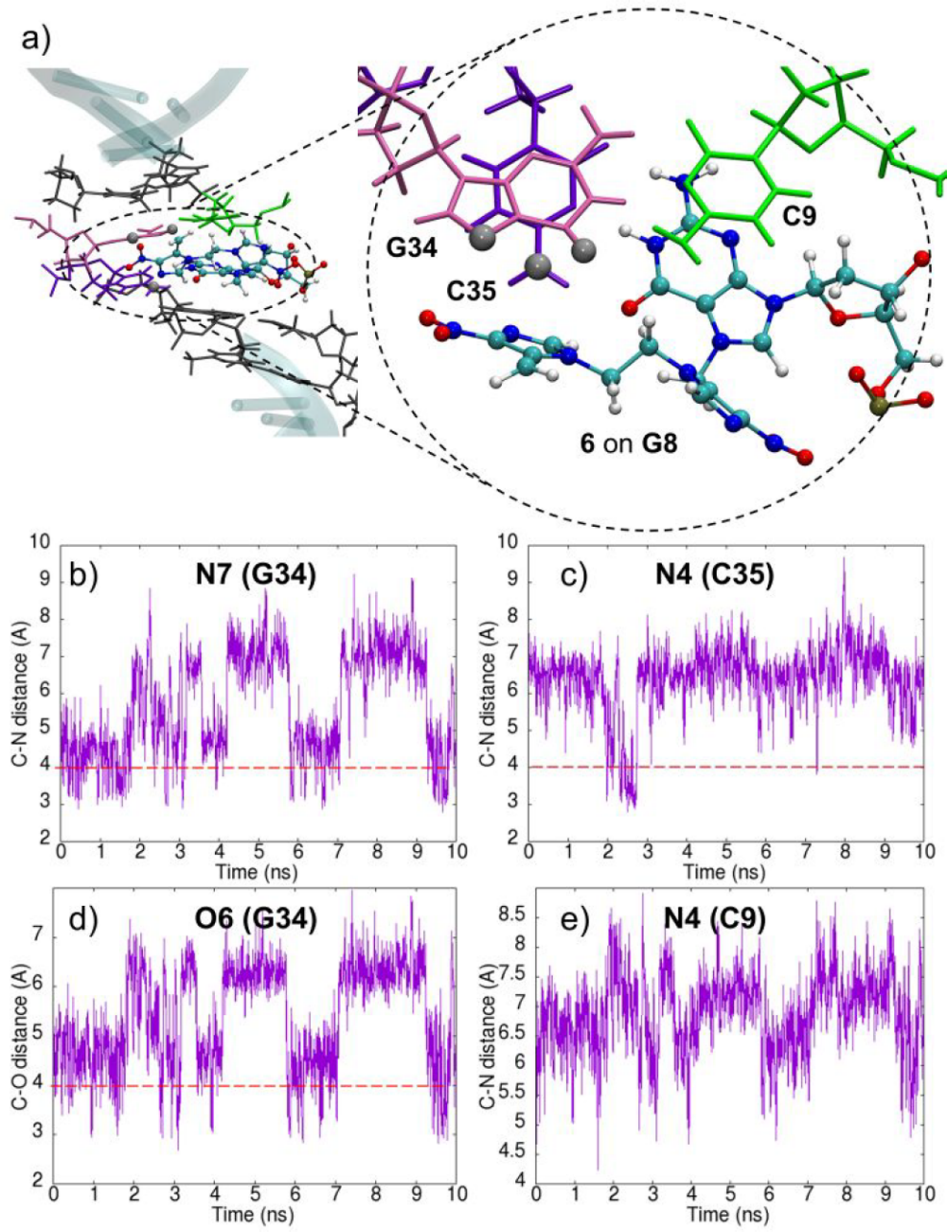
(a) Snapshot extracted from the classical MD simulation (10 ns) for the modified DNA with species **6** on **G8**. Adenine and thymine nucleobases are shown in gray. The G8(**6**)C9 step is shown in detail at the right-hand side. C9 is shown in light green, G34 in magenta, and C35 in violet. The O6 and N7 positions of G34 and the N4 position of C35 are highlighted as gray balls. Relevant atom–atom distances between the carbocation of **6** and the surrounding bases G34, C35, and C9 are shown in panels b–e. Horizontal red dashed bars placed at distances of 4 Å highlight close atom–atom proximities.

**Scheme 4:**
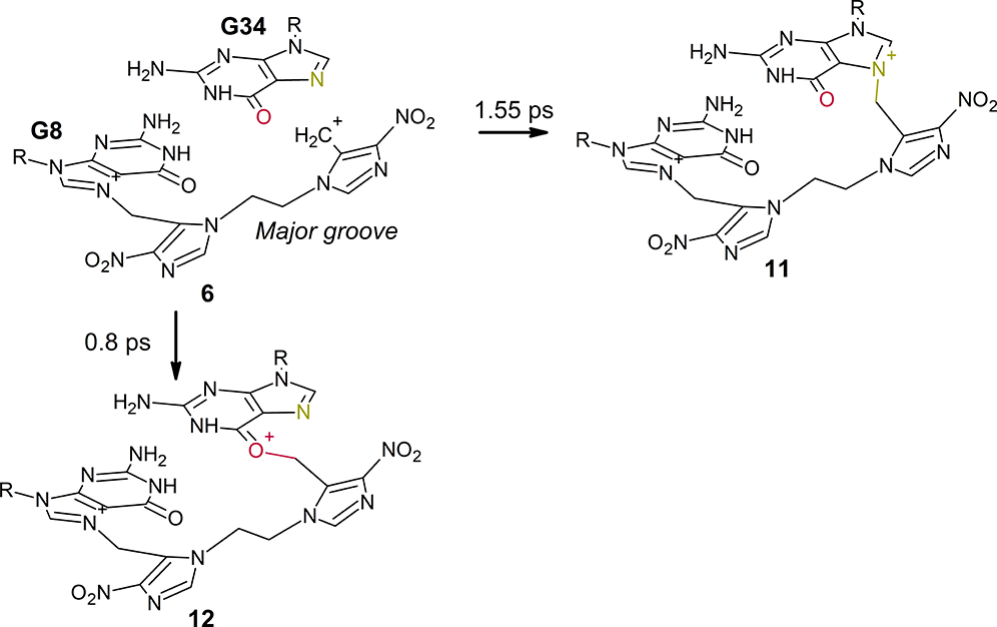
Formation of the DNA ICL Products **8** and **9** between G8 and G34 R = DNA.

The interactions with reactive hotspots over time have been studied by building a model of **6** in which the modified nucleotide lies at G8 position, as shown in Figure 5a. Note that the GC step composed by G8C9 is surrounded by AT base pairs, colored in gray. Analysis of the distances between the nearby N and O reactive positions and the carbocation of **6** (Figure 5b–e) indicate that the complementary base to G8, C35, is accessible to form an ICL (product **7**, Scheme 2). This is not surprising considering the length of the two nitroimidazole rings linked through the ethyl bridge. The most accessible reactive positions however correspond to the N7 and O6 positions of G34, as shown in Figure 5b,d, respectively. Reactions at these positions lead to the ICL products **11** and **12**, as illustrated in Scheme 4. Considering also that the reactions with these positions are kinetically fast, as previously demonstrated in Table 4, the formation of the ICL between the two guanine molecules in a GC step is deemed as one the most favorable reaction channels in the DNA major groove.

Among the major groove positions either at adenine or thymine, the N7 position of the former should be considered as the most reactive site given its high exergonicity (Table 3 and Figure S13). However, in the lesion **6** at G8, the N7 positions of A10 and A36 are not accessible within the simulation time (10 ns), whereas the opposite C35 and the contiguous G34 are readily available. Note also that the π-stacked cytosine nucleobase to G8, i.e. C9, is also less accessible (Figure 5e). This indicates that the formation of an intrastrand cross-link is unlikely as compared to the reaction between the two antiparallel DNA strands.

In contrast to the major groove, the minor groove reactivity is dominated by adenine and thymine, because the N3 position of guanine is less nucleophilic (Table 3). The efficient minor groove interaction of **2**, in conjunction with the highly exergonic reaction with the N3 position of adenine (Tables 3 and 4), make the formation of **8** (Scheme 3) a very favorable process. The subsequent photoinduced release of mechlorethamine produces **9**.

**Figure 6:**
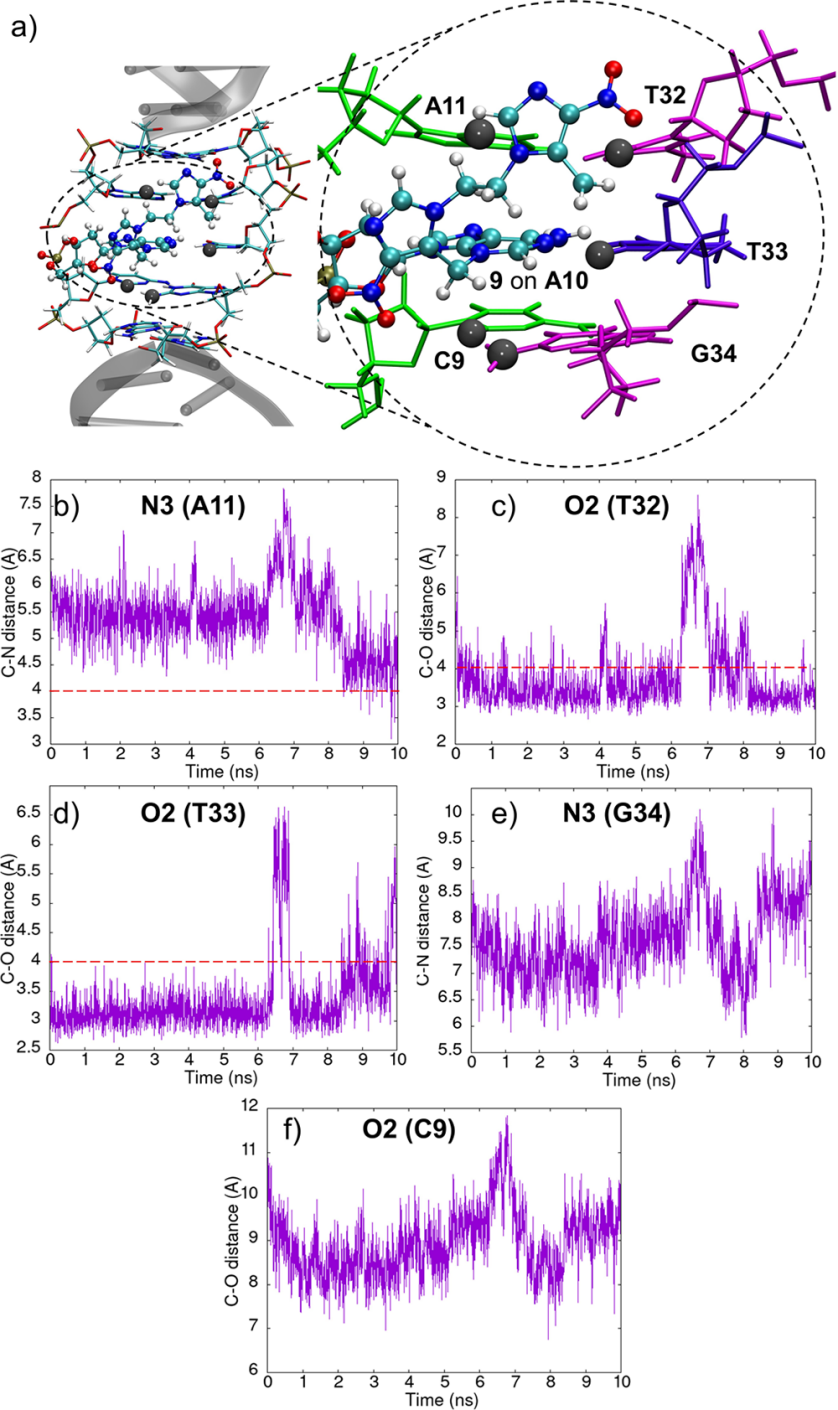
(a) Snapshot extracted from the classical MD simulation (10 ns) for the modified DNA with species **9** on A10. Adenine and thymine nucleobases are shown in gray. The C9A10(**9**)A11 steps are shown in detail at the right-hand side. Relevant atom–atom distances between the carbocation of **9** and the surrounding bases C9, A11, T32, T33, and G34 are shown in panels b–f. Horizontal red dashed bars placed at distances of 4 Å highlight close atom–atom proximities.

Figure 6 quantifies the close contacts between the carbocation lesion **9** on A10 and the most reactive positions of nearby nucleobases. The O2 positions of the opposite T33 and its adjacent T32 nucleobases are clearly the most available sites (Figure 6c,d). The adjacent positions to the lesion, i.e. C9 and A11, are much less available (Figure 6b,f). Only A11 exhibits certain close contact with the carbocation, even though to a much less extent than the thymine molecules. The N3 position of G34, besides being less reactive than the O2 site of thymine, is not available (Figures 6e). These data strongly support that the most probable reactions of **9** and DNA take place with thymine to form, for example, **10** (Scheme 3). It is therefore reasonable to conclude that adenine and thymine dominate the DNA minor groove reactivity. The O2 position of cytosine should exhibit a similar reactivity to the O2 position of thymine (Table 3); however, the complementarity between adenine and thymine and the high reactivity of adenine decreases the relevance of cytosine in the minor groove reactivity, although ICLs between adenine and cytosine are probably sufficiently fast to occur.

**Figure 7:**
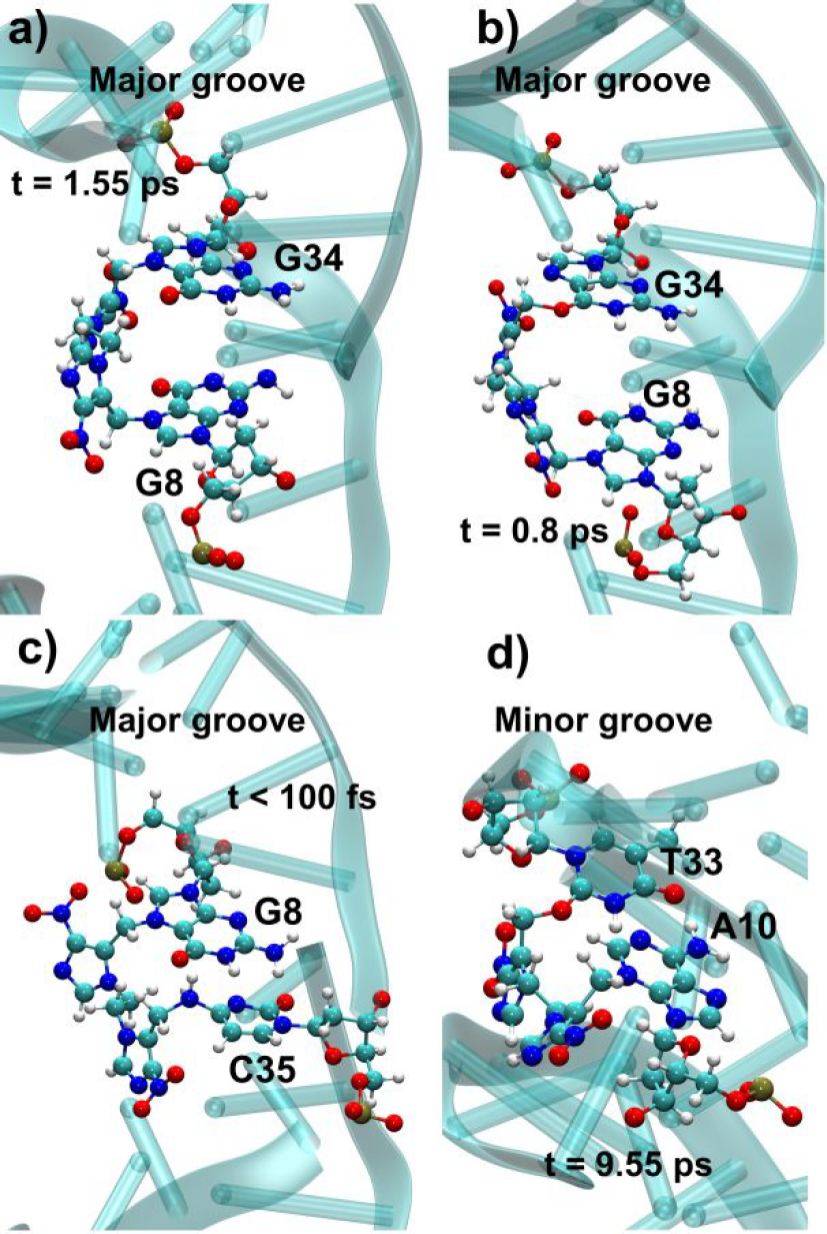
DNA ICL products formed with QM/MM dynamics among (a) G8 and G34 at N7 position (**11**), (b) G8 and G34 at N7 and O6 positions, respectively (**12**), (c) G8 and C35 at N7 and N4 positions, respectively (**7**), and (d) A10 and T33 at N3 and O2 positions, respectively (**10**).

The reactivity between **6** (at G8) and **9** (at A10) and the available positions in the DNA surroundings have been explored by using the same QM/MM protocol used to study the reactivity of **2** toward DNA in Table 4. ICLs between **6** at G8 and G34, both at N7 and O6 positions of the latter nucleobase (species **11** and **12**, respectively, Scheme 4), have been observed at the picosecond scale, as shown in Figure 7a,b. The reaction of **6** with its complementary Watson–Crick base pair C35 at the N4 position, to yield the ICL product **7** (Scheme 2 and Figure 7c), has been also observed in the QM/MM simulations. Similarly, the reaction between **9** at A10 and the O2 position of T33 to yield **10** (Scheme 3) occurred after 9.55 ps (Figure 7d).

Globally, these observations clearly imply that the limiting step for the formation of the second DNA-photosensitizer covalent bond is the mechlorethamine photorelease. Close interactions between **6** and G34 and between **8** and T32/T33 take place in less than 1 ns (Figures 5 and 6), while the subsequent reaction kinetics are most probably barrierless and limited by diffusion, taking place at the picosecond time scale, as observed in Figure 7. The formation of **7** is expected to be less favorable as compared to **11** and **12** because close interactions between **6** and the susceptible N4 atom of C35 are less frequent (Figure 5c) and the N4 position of cytosine is far less reactive than the N7 and O6 sites of guanine (Table S6).

The preference for the N7 position of guanine and adenine has been traditionally ascribed to the Maxam and Gilbert reactions,^63−65^ in which the ICLs at the N7 position of purines cleave upon treatment with piperidine. The N7 position of guanine is also largely known to be the preferred reaction site for nitrogen mustards such as mechlorethamine^66^ and of traditional anticancer drugs such as *cis*-platin.^67^ To the best of our knowledge, the relevance of the O6 site of guanine has not been documented until now.

### From Light Absorption to DNA Interstrand Cross-Linking in Biological Media: Spatial and Temporal Resolution of the Global Process

**Figure 8:**
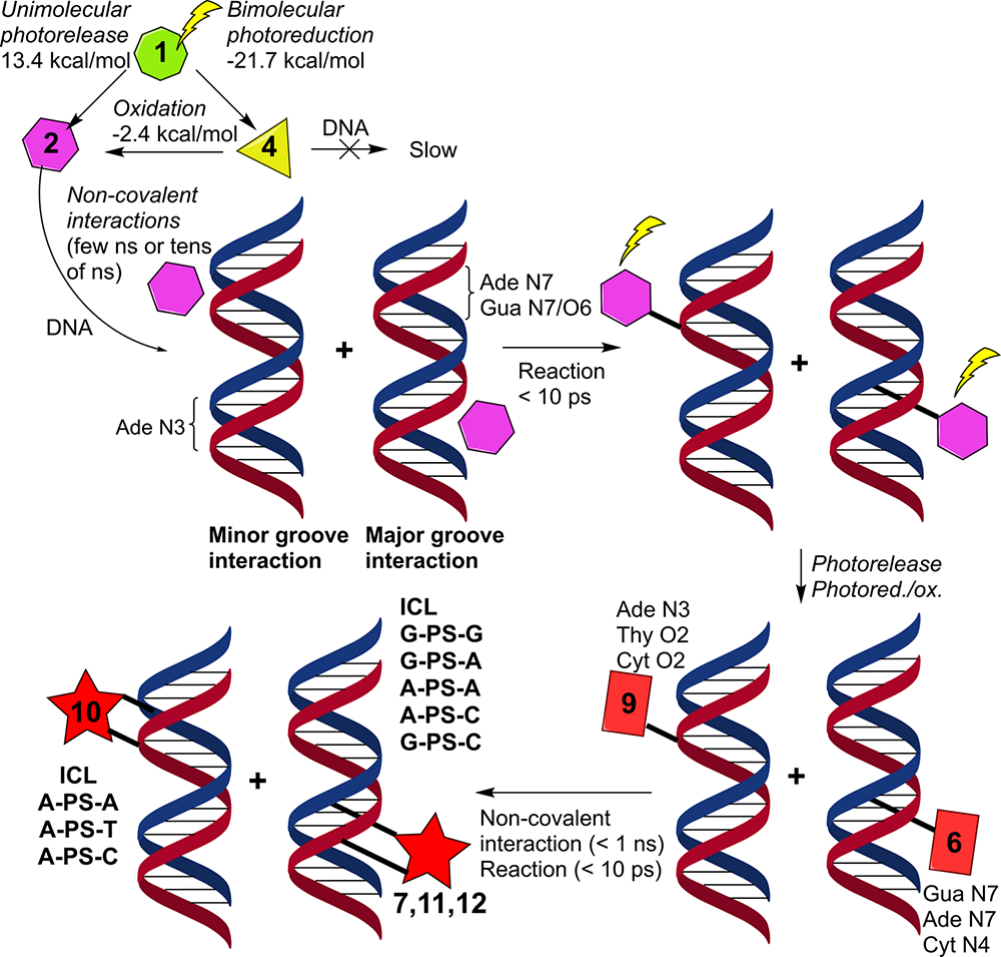
Schematic representation of the temporal and spatial scales for the DNA ICL mechanism revealed by the combination of QM, classical MD, and hybrid QM/MM simulations performed in this work.

The global mechanism extracted from the present simulations is shown in Figure 8. The bottlenecks are the light induced releases of the two mechlorethamine leaving groups through the unimolecular or bimolecular mechanisms. Both **2** and **4** species show favorable noncovalent major and minor groove interactions with DNA (in a few nanoseconds or tens of nanoseconds), while the reactivity of **2** is much faster as compared to **4**. In 10 ps, ca. 25% of **2** reacted with DNA, both in major or minor grooves (Table 4). We have also identified the most reactive nucleobases and the regioselectivity within a given nucleobase. The N7 position of purines (and O6 of guanine) show the kinetically fastest reactions in the major groove, whereas the minor groove is governed by the N3 position of adenine. Bearing this in mind and considering also the reactivity of pyrimidines and the spatial reach of the two fused nitroimidazole rings, which spans the Watson–Crick base pair with the covalent lesion (for instance **6** and **9**) and the adjacent DNA step, GC, GT, and AT steps are identified as the most relevant hotspots for ICL formation, independently from the DNA sequence. Although other ICL products are possible, we highlight here only the most kinetically competitive routes. For instance, GG or CC tuples are deemed less reactive than GC, GT, or AT steps. In GG and CC, the most reactive nucleobases (guanine) are stacked, therefore, the corresponding ICL formation is slower as in GC, in which the two guanine nucleobases are placed in opposite strands. The same conclusion applies to TT or AA steps.

## Conclusions

Extensive and detailed multiscale calculations have rationalized the spatial and temporal scales of the ICL formation in double stranded B-DNA. The molecular mechanisms behind light absorption and C–N cleavage of the photosensitizer have been delineated at the electronic level by using quantum chemistry, the noncovalent interactions have been fully characterized by using all-atom MD at the microsecond scale, and the complex regioselectivity of covalent bonding with DNA has been resolved through multiple QM/MM simulations. Among all events considered in this work, the generation of the reactive carbocation **2** is the rate-limiting step, although the combination of persistent interactions with DNA and the photoreduction/oxidation reactions mediated by guanine likely facilitates this process. Once formed, **2** efficiently interacts with major and minor DNA grooves.

The fastest major groove reactions take place with the N7 and O6 positions of guanine, the N7 position of adenine, and the N4 position of cytosine. These are barrierless or almost barrierless processes. In the minor groove, adenine shows the fastest reactivity. As a result, GC, GT, and AT steps have been identified as the most favorable hotspots for ICL. This sequence effect and the high reactivity of the O6 position of guanine, not reported until now, should be taken into account in the design of new photosensitizers, for example, when deciding the molecular length of the active species and when tuning the electrophilic character of its reactive site. The molecular length is particularly crucial, because it has been clearly demonstrated that the cross-linking can take place involving several Watson–Crick base pairs. Subsequently, longer and flexible agents will link distant nucleobases.

The DNA models studied in this work simulate the experiments with short DNA sequences in solution performed by Han et al.^42^ However, it shall be noted that in actual eukaryote cells the DNA is packed around histones forming nucleosomes, a disposition that will limit the accessibility of the photosensitizer to the coiled DNA. A similar regioselectivity toward the different nucleobases is nevertheless expected because the nucleophilicity is an intrinsic property of each atomic position. Because a single unrepaired ICL lesion is sufficient to arrest the DNA biological functioning,^24^ a small portion of “free” DNA or RNA reachable by the photosensitizer may be sufficient to deliver DNA damage with the present approach. This is particularly true in the replication cycle or in expressed genes, in which DNA is less strictly packed to allow replication/translation. On the other hand, well-known DNA–metal interactions^68−71^ may influence the reactivity of **2** with DNA. A thorough scrutiny of these environment effects falls out of the scope of the present research and would deserve an independent study.

The present work provides a detailed and innovative description of DNA cross-linking, a crucial phenomenon in biology and medicine, and settles a computational approach to study these important events far beyond the static description of chromophore models.

## Computational Methodology

The different levels of theory adopted in each subsection were chosen according to the respective validations, because the nature of the chemical processes studies in each subsection is diverse (light absorption and photochemical reactivity, photoredox reactions, and DNA reactivity) and have different methodological requirements.

### Multiconfigurational Calculations

The complete-active-space self-consistent-field (CASSCF) method^72^ as implemented in the OpenMolcas software^73^ was used to build the multiconfigurational wave functions of **1**. The Franck–Condon geometry was optimized using the B3LYP/6-31G* method.^74,75^ Here 12 singlet states and 10 triplet states were demanded in the respective state-average (SA) procedures. The necessary electronic dynamic correlation was calculated with the CASPT2 method^76,77^ on top of the SA-CASSCF wave functions. The IPEA shift^78,79^ was set to 0.0 au (original zeroth-order Hamiltonian), and an imaginary level shift of 0.2 au was used to avoid the presence of intruder states. Oscillator strengths and spin–orbit couplings were computed using the SA-CASSCF wave functions and the CASPT2 energies, as described in previous works.^80−85^ The ANO-S-VDZP basis set was used in all multiconfigurational determinations.

### Coupled-Cluster Calculations

For benchmark purposes, domain-based local pair-natural orbital coupled cluster singles, doubles, and perturbative triples (DLPNO-CCSD(T)) calculations were performed on top of the DFT/B3LYP optimized structures (see below) for the addition of **4** to the C5 and C6 positions of thymine. The employed basis set was 6-311+G(2df,2pd). All DLPNO-CCSD(T) calculations were performed with the ORCA 5.0 software.^86^

### DFT Calculations

The DFT/B3LYP functional in combination with the 6-31G* basis set and its TD-DFT extension^87^ was used to compute the absorption properties of **1** and **4**, as validated by CASPT2 determinations. Natural transition orbitals (NTOs)^51,52^ were computed with the Chemissian software.^88^ Solvent effects (water) were considered using the polarizable continuum model (PCM). As indicated in Table 1, the (photo)redox reactions were tackled with the M06-2X/6-31++G(d,p) method since previous DNA-related works showed that this level of theory properly describes these types of reactions, as compared to experimental observations.^56,57^ The B3LYP-PCM/6-31G*method was initially chosen to calculate the activation energy and energy difference between the reactants and products of the radical addition of **4** to the C5 and C6 positions of thymine. Frequency calculations were carried out on top of the converged minima and TSs to check the absence of any negative eigenvalue of the Hessian or the presence of only one, respectively. Intrinsic reaction coordinate (IRC) paths confirmed the connectivity between reactants, TSs, and products. According to the performed benchmarks, the reactivity of **4** (small QM model, see text) with the four DNA nucleobases was described with M06-2X/6-31G* (Table S7). Gibbs free energies at 298 K and 1 atm at the stationary points were computed at the same level of theory. The barrierless paths of the reactivity of **2** were described through relaxed scans of the reaction coordinates. The Δ*G* values of Table 3 were computed using the isolated small model of **2** and the corresponding nucleobases as a reference. The basis set superposition error (counterpoise procedure) was computed for the product of **2** at the O2 position of thymine and cytosine and at the C5 position of guanine and adenine, respectively. The same value was used to correct the Gibbs energies of the rest of reaction products belonging to the same nucleobase. All DFT and TD-DFT calculations were performed with the Gaussian 16 software package.^89^

### Classical Molecular Dynamics

The initial structure of the B-DNA double strand with sequence 5′-TTGCAATGCAAGTAATTAAAG-3′ (Figure 1) was built with the NAB utility^90^ available in AmberTools.^91^ The species **1**, **2**, and **4** were randomly positioned next to the DNA strand, in the majority of cases close to a nucleic acid major groove (see Figure S29). One run started with **4** intercalated between two base pairs, although this configuration is highly unstable, as mentioned. The systems were later placed at the center of a truncated octahedral box of TIP3P^92^ water molecules in which the minimum distance between the solutes and the edge of the box was 10 Å. The systems were neutralized by adding Na^+^ cations, giving rise to a final [Na^+^] of ∼130 mM, only slightly above the 1–100 mM physiological concentrations reported for Na^+^, K^+^, and Mg^2+^.^69^ DNA was described using the parm99 force field^93^ including bsc1 corrections.^94^ The force field parameters for the photosensitizers **1**, **2**, and **4** were obtained from GAFF using standard protocols. Geometry optimizations were performed with the B3LYP/6-311G(d) method. The restricted electrostatic potential (RESP) method was used to calculate the atomic charges, obtained with the Hartree–Fock (HF) method in conjunction with the 6-31G* basis set. The modified nucleobases **6** and **9** were initially optimized also using the B3LYP/6-311G(d) level of theory, whereas the electrostatic potential was computed with the HF/6-31G* method. **6** and **9** were parametrized based on the Amber force field using also the RESP method to calculate the atomic charges. Structures and point charges of residues **1**, **2**, **4**, **6**, and **9** used for the parametrization can be found in the Supporting Information. The systems **1** + DNA, **2** + DNA, and **4** + DNA were minimized through 6000 steps of the steepest descent algorithm followed by 6000 steps of the conjugated gradient algorithm. Thermalization at 300 K was performed in 200 ps in the NVT ensemble. Six microseconds of production dynamics (6 trajectories of 1 μs each, see Table 2) were carried out in the NPT ensemble. Coordinates and velocities were written into the trajectory files at time intervals of 40 ps. The pressure was set to 1 atm and kept constant using the Monte Carlo barostat, while temperature conservation was ensured by employing Langevin dynamics. The same protocol was applied to the modified DNA systems **6** and **9**, except the NPT production run, which was propagated for 10 ns. One trajectory was run for **6** and another one for **9**. All MD simulations were performed under periodic boundary conditions and using particle mesh-ewald (PME) with a cutoff of 9.0 Å and a time step of 1 fs. All classical MD simulations were performed with the Amber 20 software package.^91^ Results were analyzed with the visual molecular dynamics (VMD) program^95^ and with the Cpptraj^96^ tool included in the Amber 20 package. In summary, three noncovalent DNA/photosensitizer (**1**, **2**, and **4**; one, two, and three replicas, respectively) and two covalent DNA/photosensitizer (**6** and **9**, one replica each one) systems were simulated.

### QM/MM Simulations

QM/MM molecular dynamics making use of the electrostatic embedding were used to explore the reactivity of **2** with DNA in a water box. Initial conditions (atom coordinates and velocities) were extracted from the classical MD trajectories. Newton’s equations of motion were solved with a time step of 1 fs. The QM partition was composed of the nitroimidazole ring (including the carbocation) and the corresponding DNA nucleobase, i.e. the small model in Figure S12, and was treated with the M06-2X/6-31G* method as implemented in the ORCA 5.0 software. The QM method validation is described. The dynamics were performed using the Amber/ORCA QM/MM interface. A cutoff of 9.0 Å was employed for all QM/MM interactions. The competition between the most reactive pathways was assessed by extending the number of runs starting from different initial conditions. Snapshots were selected from the classical MD trajectories with sufficiently separated time intervals to avoid the overrepresentation of a given conformation, as pointed out in detail in Figures S14–S28. Note that the frames of the classical MD trajectories are separated from each other by 40 ps. The total QM/MM simulation time in this work largely exceeds 500 ps.

## Supporting Information Available

CASPT2 vertical absorptions and oscillator strengths, CAS active space, TD-DFT electronic spectra, minimum energy path for the dissociation of **3**, RMSD values of the MD simulations, histograms of noncovalent energies and photosensitizer-DNA distances, validation of the DFT functional and basis set, additional DFT results, QM/MM sampling of the classical MD runs, and initial structures for the MD simulations (PDF)

## Notes

VMD, OpenMolcas, Orca 5, and the CPU versions of Amber 20 (used to compute QM/MM dynamics) and AmberTools are publicly available and free of charge. Other widely known programs such as Gaussian 16 and the GPU implementation (CUDA version) of Amber 20 are commercially available. The computational methodology section and the Supporting Information provide details to reproduce all computations.
