# Supplementary material for: Spatial and Temporal Resolution of the Oxygen-Independent Photoinduced DNA Interstrand Cross-Linking by a Nitroimidazole Derivative
Source: J Chem Inf Model. 2022 Jun 30;62(13):3239–52. doi: 10.1021/acs.jcim.2c00460 (PMC9277591; doi:10.1021/acs.jcim.2c00460)
Supplement: Supplementary file 1 — ci2c00460_si_001.pdf [file ci2c00460_si_001.pdf]

SUPPORTING INFORMATION FOR:

Spatial and Temporal Resolution of the Oxygen-Independent Photoinduced DNA Inter-Strand Cross-Linking by a Nitroimidazole Derivative

Abdelazim M. A. Abdelgawwad,[a] Antonio Monari,[b,c] Iñaki Tuñón[a] and Antonio Francés-Monerris[a]*

a) Departament de Química Física, Universitat de València, 46100 Burjassot, Spain.

*E-mail: antonio.frances@uv.es

b) Université Paris Cité, CNRS, ITODYS, F-75006 Paris, France.

c) Université de Lorraine and CNRS, UMR 7019 LPCT, F-5400, Nancy, France.

Table S1. Spin-orbit free vertical absorption energies (E_{abs}) in eV and associated oscillator strengths (*f*) for species **1** computed with the CASPT2 method. The active space used is CAS(12,12). 12 singlet states and 10 triplet states were demanded in the SA-CASSCF procedure. Bright states are highlighted in bold.

**State**	$\boldsymbol{E_{abs}}$	***f***	**State**	$\boldsymbol{E_{abs}}$
$\mathbf{S_2}$	**4.87**	**0.3048**	$\mathbf{T_2}$	3.27
$\mathbf{S_3}$	**4.87**	**0.1325**	$\mathbf{T_3}$	3.32
$\mathbf{S_4}$	5.55	0.0051	$\mathbf{T_4}$	3.95
$\mathbf{S_5}$	5.67	0.0041	$\mathbf{T_5}$	3.98
$\mathbf{S_6}$	5.85	0.0085	$\mathbf{T_6}$	5.41
$\mathbf{S_7}$	5.87	0.0089	$\mathbf{T_7}$	5.43
$\mathbf{S_8}$	6.31	0.0000	$\mathbf{T_8}$	6.48
$\mathbf{S_9}$	6.75	0.0011	$\mathbf{T_9}$	6.72
$\mathbf{S_{10}}$	6.88	0.0000	$\mathbf{T_{10}}$	6.81
$\mathbf{S_{11}}$	7.47	0.0000		
$\mathbf{S_{12}}$	8.38	0.0000		

Table S2. Spin-orbit vertical absorption energies (E_{abs}) in eV and associated oscillator strengths (f) for species **1** computed with the CASPT2 method. The active space used is CAS(12,12). 12 singlet states and 10 triplet states were demanded in the SA-CASSCF procedure. All computed singlet-triplet spin-orbit couplings are smaller than 0.5 cm^{-1}. Bright states are highlighted in bold.

State	E_{abs}	f
SO_2	3.27	0.0000
SO_3	3.27	0.0000
SO_4	3.27	0.0000
SO_5	3.32	0.0000
SO_6	3.32	0.0000
SO_7	3.32	0.0000
SO_8	3.95	0.0000
SO_9	3.95	0.0000
SO_{10}	3.95	0.0000
SO_{11}	3.98	0.0000
SO_{12}	3.98	0.0000
SO_{13}	3.98	0.0000
SO_{14}	**4.87**	**0.3048**
SO_{15}	**4.87**	**0.1325**
SO_{16}	5.41	0.0000
SO_{17}	5.41	0.0000
SO_{18}	5.41	0.0000
SO_{19}	5.43	0.0000
SO_{20}	5.43	0.0000
SO_{21}	5.43	0.0000
SO_{22}	5.55	0.0051
SO_{23}	5.67	0.0041
SO_{24}	5.85	0.0085
SO_{25}	5.87	0.0089
SO_{26}	6.31	0.0011
SO_{27}	6.48	0.0000
SO_{28}	6.48	0.0000
SO_{29}	6.48	0.0000
SO_{30}	6.72	0.0000
SO_{31}	6.72	0.0000
SO_{32}	6.72	0.0000
SO_{33}	6.75	0.0000
SO_{34}	6.81	0.0000
SO_{35}	6.81	0.0000
SO_{36}	6.81	0.0000
SO_{37}	6.88	0.0000
SO_{38}	7.14	0.0000
SO_{39}	7.14	0.0000
SO_{40}	7.14	0.0000
SO_{41}	7.47	0.0000
SO_{42}	8.38	0.0000

Table S3. Vertical absorption energies (E_{abs}) and wavelengths (λ_{abs}) and associated oscillator strengths (*f*) for species **1** computed with TD-B3LYP method. Bright states are highlighted in bold.

State	E_{abs}	λ_{abs}	*f*
S_2	3.68	336.7	0.0000
S_3	3.71	333.8	0.0000
S_4	4.15	298.8	0.0002
S_5	4.16	298.0	0.0001
S_6	4.36	284.6	0.0006
S_7	4.50	275.8	0.0002
S_8	4.57	271.0	0.0005
S_9	4.67	265.2	0.0059
S_{10}	4.71	263.5	0.0000
S_{11}	**4.80**	**258.3**	**0.0853**
S_{12}	4.84	256.2	0.0002
S_{13}	**4.85**	**255.6**	**0.0254**
S_{14}	4.88	254.2	0.0051
S_{15}	**4.89**	**253.5**	**0.0288**
S_{16}	4.92	252.0	0.0005
S_{17}	4.93	251.7	0.0009
S_{18}	**4.95**	**250.3**	**0.0890**
S_{19}	4.97	249.2	0.0104
S_{20}	5.01	247.4	0.0150

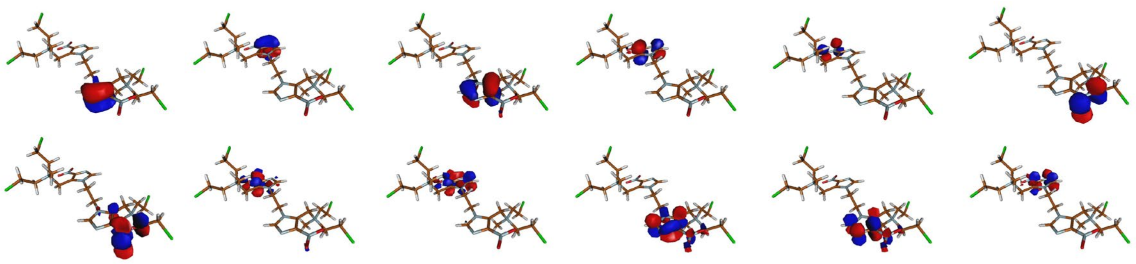

Figure S1. CAS(12,12) employed in the SA-CASSCF/CASPT2 determinations.

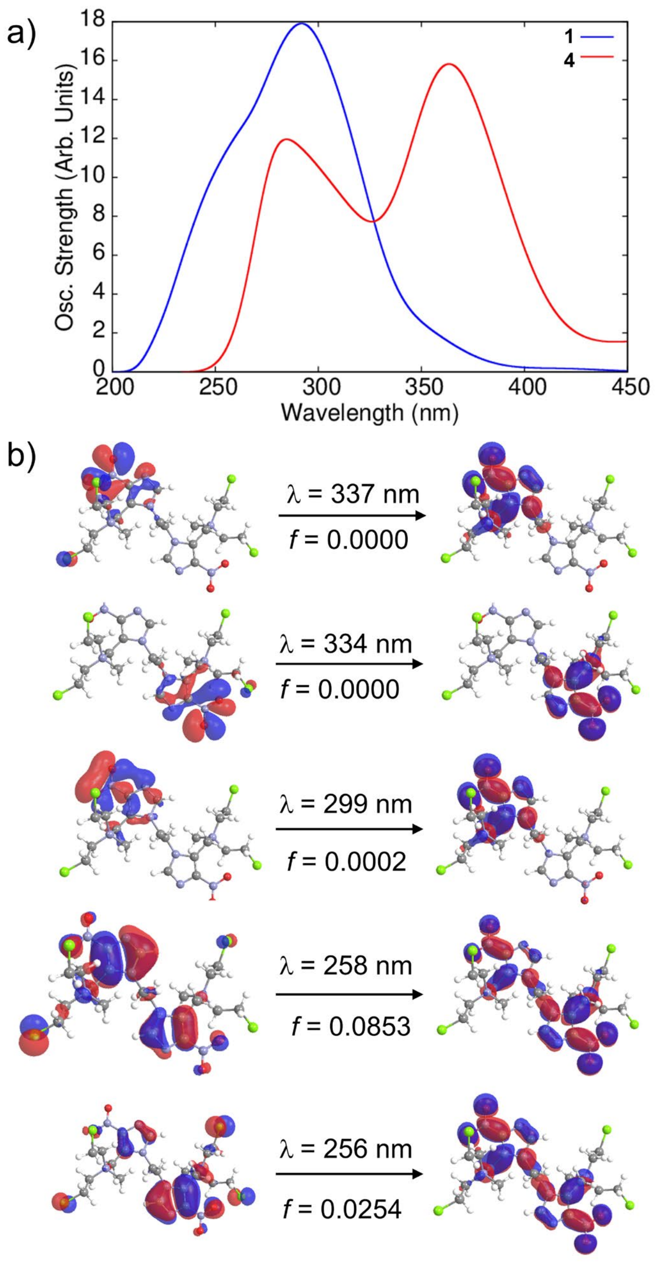

Figure S2. Absorption properties of the photosensitizer **1** and the derivative **4**. a) TD-B3LYP spectrum (first 30 singlet excited states) computed by convoluting the absorption energies and oscillator strengths of 100 structures from the Wigner distribution sampling of the Franck-Condon region for species **1** and **4**. b) Natural transition orbitals (NTOs) for the low-energy excited states (S_1-S_3), of n_{NO2},π^* nature, and for the bright states (S_{10} and S_{12}), of π,π^* nature, computed with the Chemissian software.

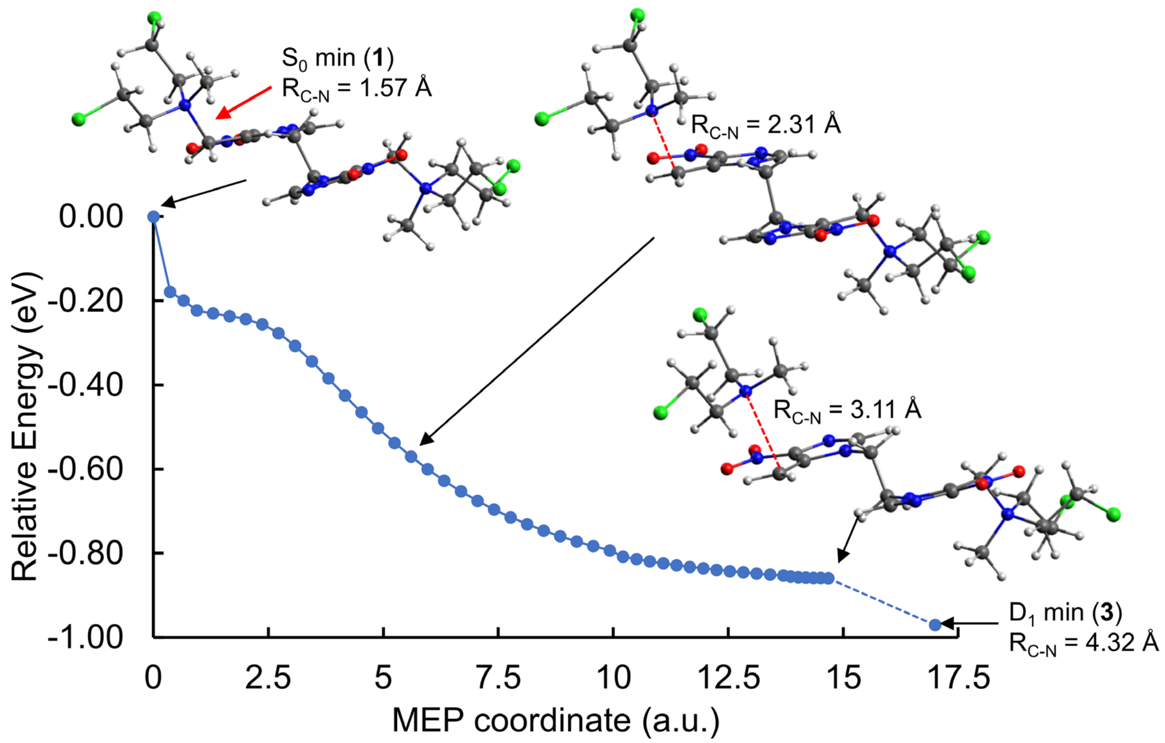

Figure S3. Minimum energy path (MEP) of **3** at the unrestricted DFT level of theory in water solution. The C-N bond breaks spontaneously as evidenced by the barrierless MEP profile.

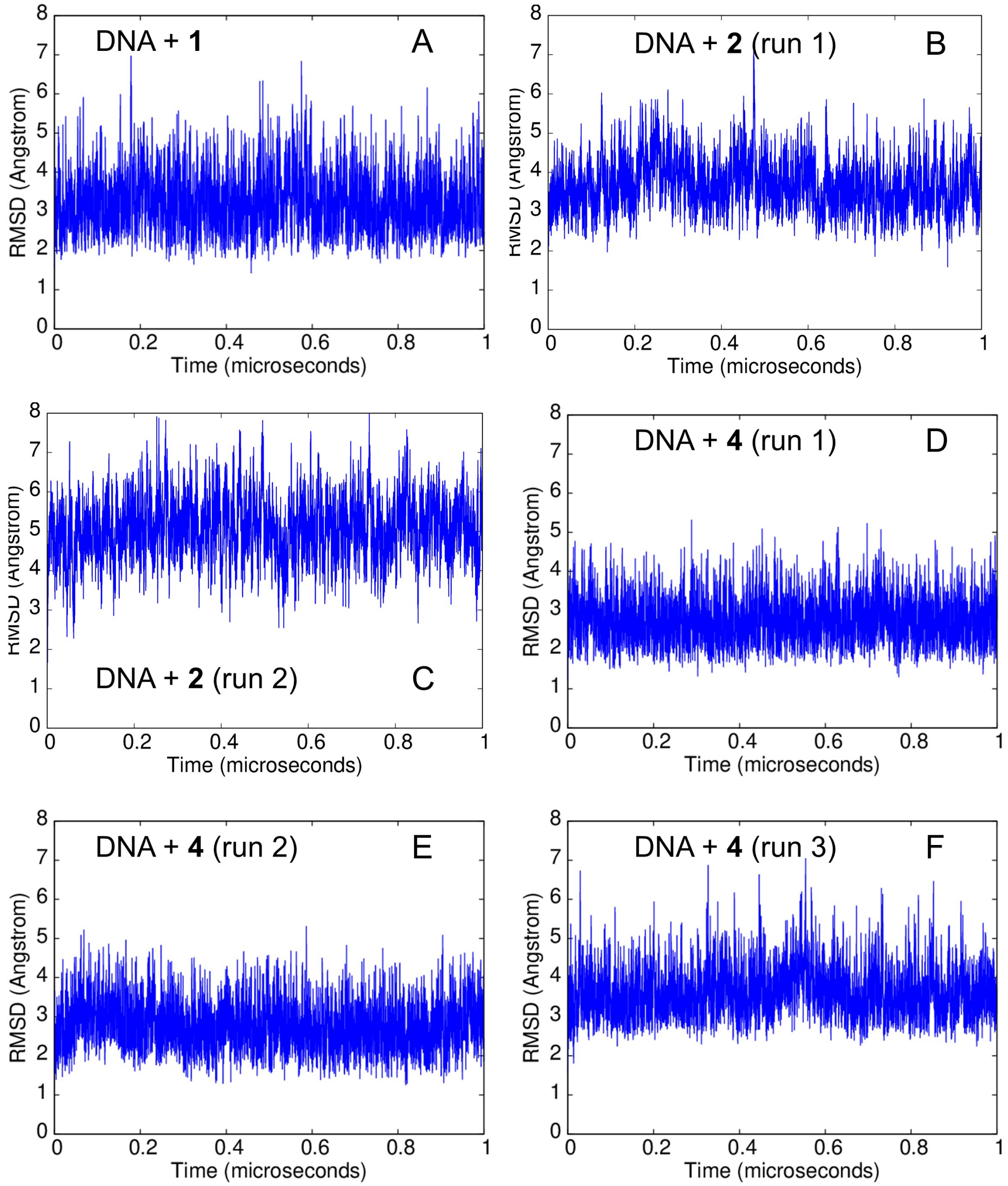

Figure S4. Root mean squared deviation (RMSD) for the double stranded DNA (sequence 5'-TTGCAATGCAAGTAATTAAAG-3') in presence of **1** (panel A) and **4** (panels B, C, and D).

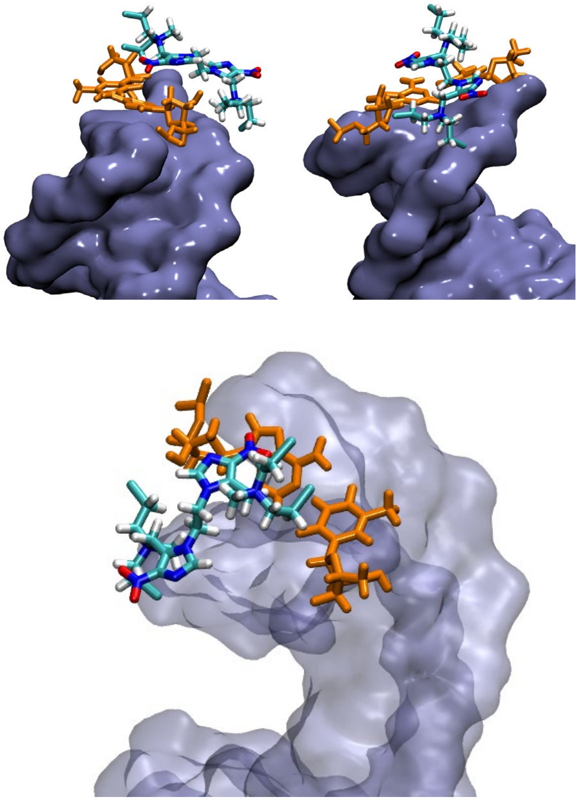

Figure S5. Snapshot of **1** (licorice representation) interacting with the T(#1)-A(#42) Watson-Crick base pair (ocre).

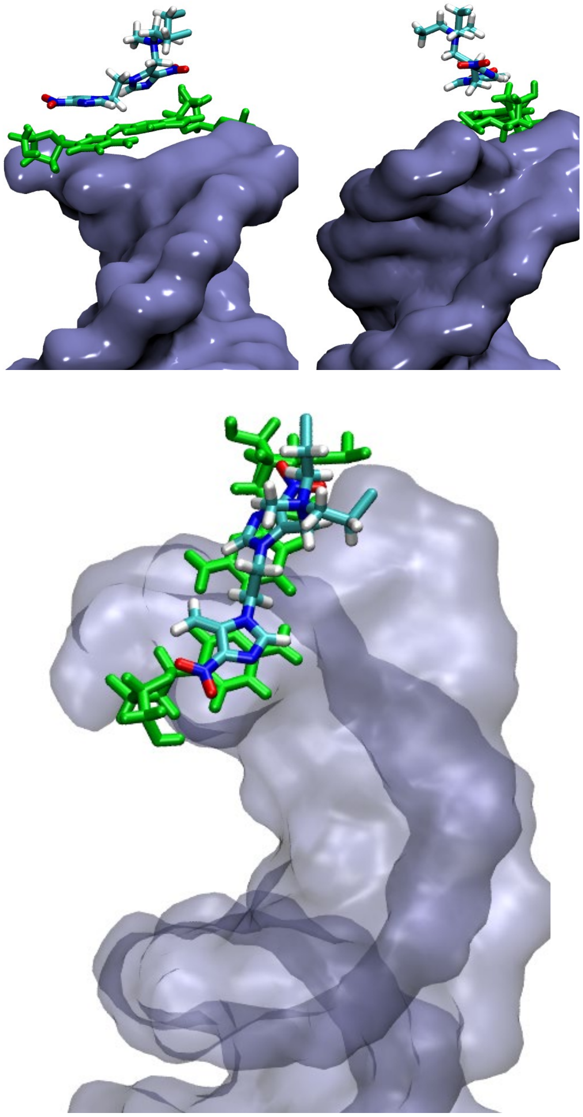

Figure S6. Snapshot of **4** (licorice representation) interacting with the G(#21)-C(#22) Watson-Crick base pair (green).

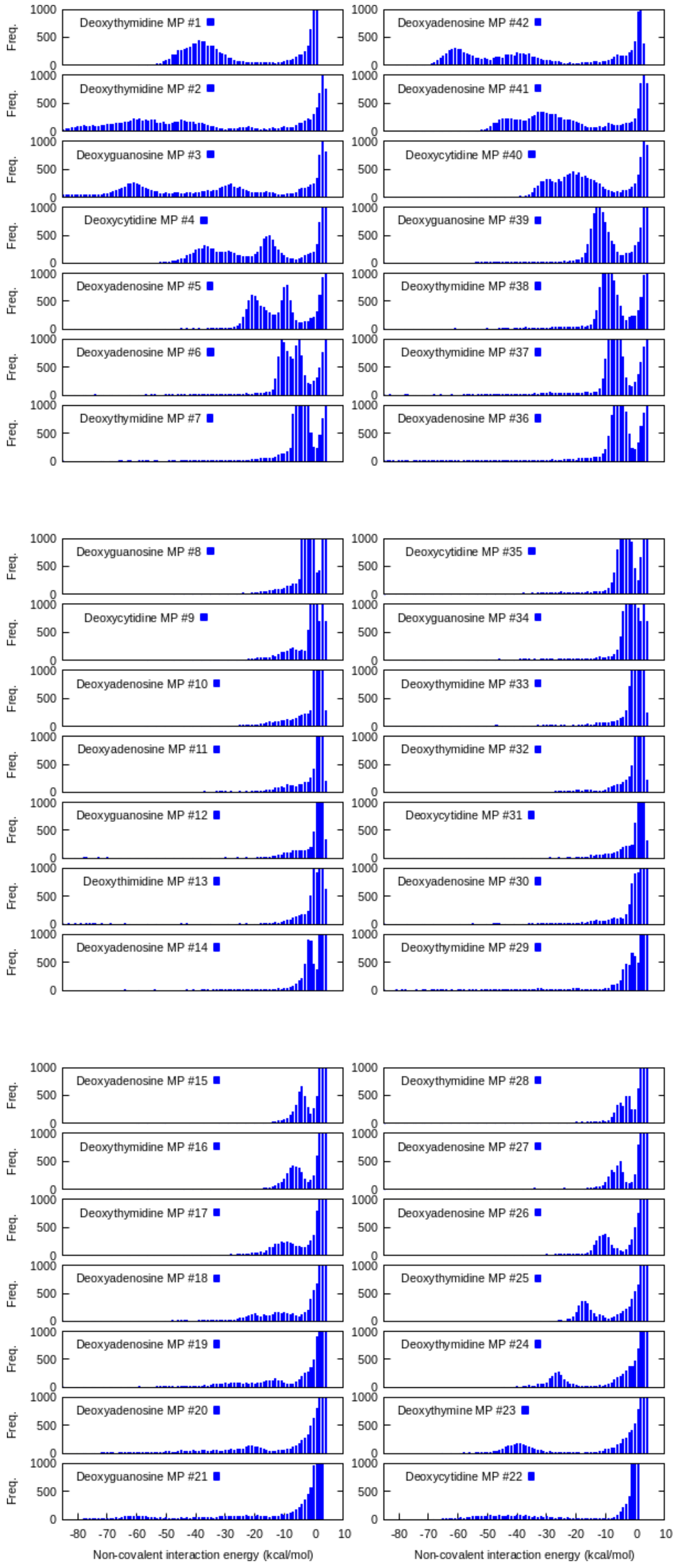

Figure S7. Non-covalent (electrostatic + van der Waals) interaction energy between **1** and the 42 nucleotides of the DNA double strand (sequence 5'-TTGCAATGCAAGTAATTAAAG-3').

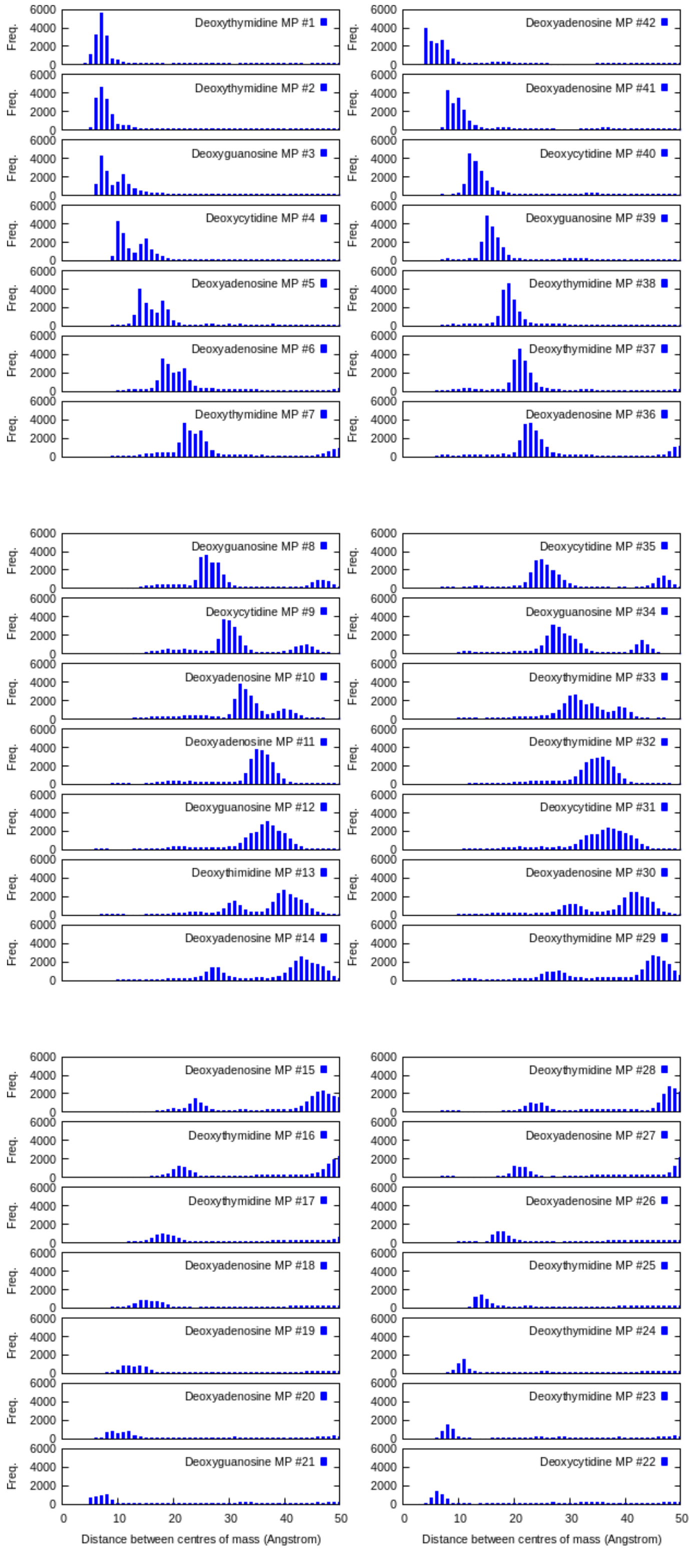

Figure S8. Histogram of the distances between the centre of mass of **1** and the centre of mass of each nucleotide (sequence 5’-TTGCAATGCAAGTAATTAAAG-3’).

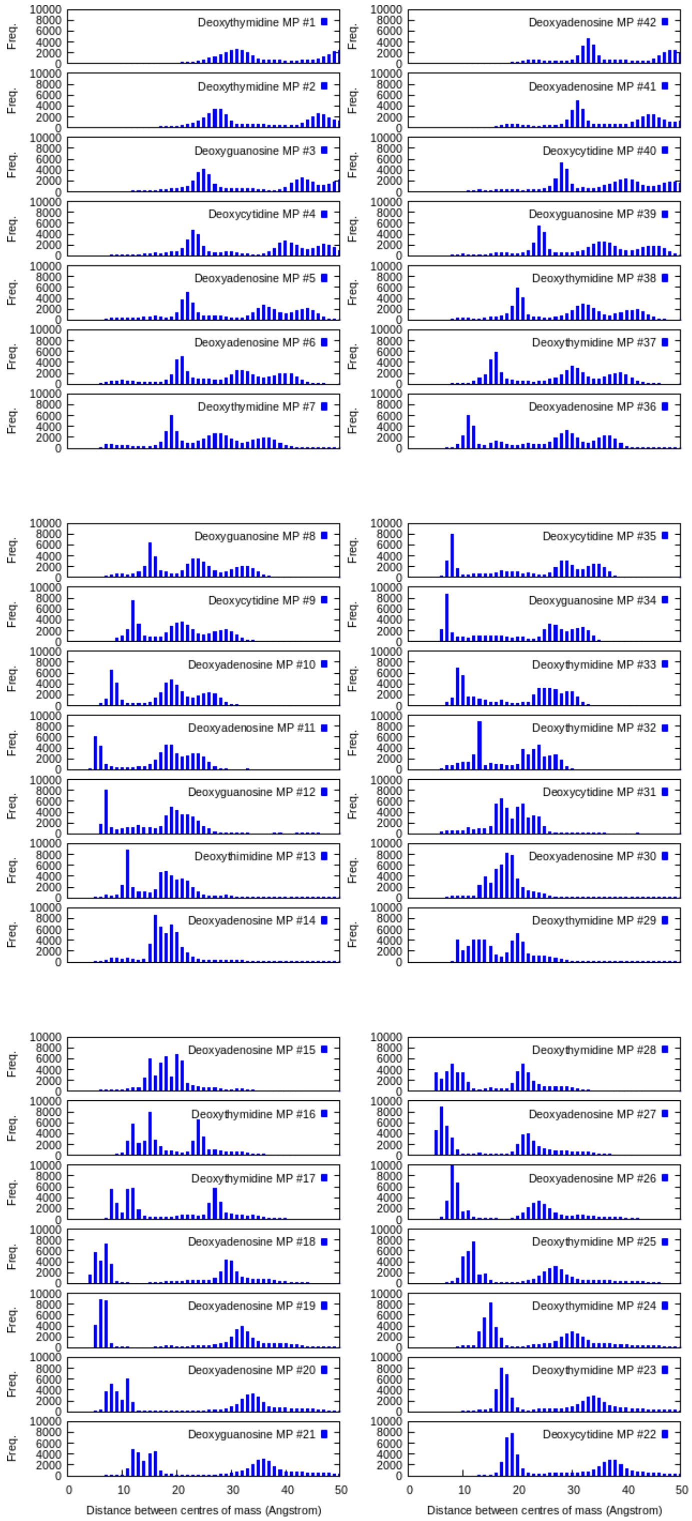

Figure S9. Histogram of the distances between the centre of mass of **2** and the centre of mass of each nucleotide (sequence 5’-TTGCAATGCAAGTAATTAAAG-3’). Data corresponds to the sum of the two MD runs.

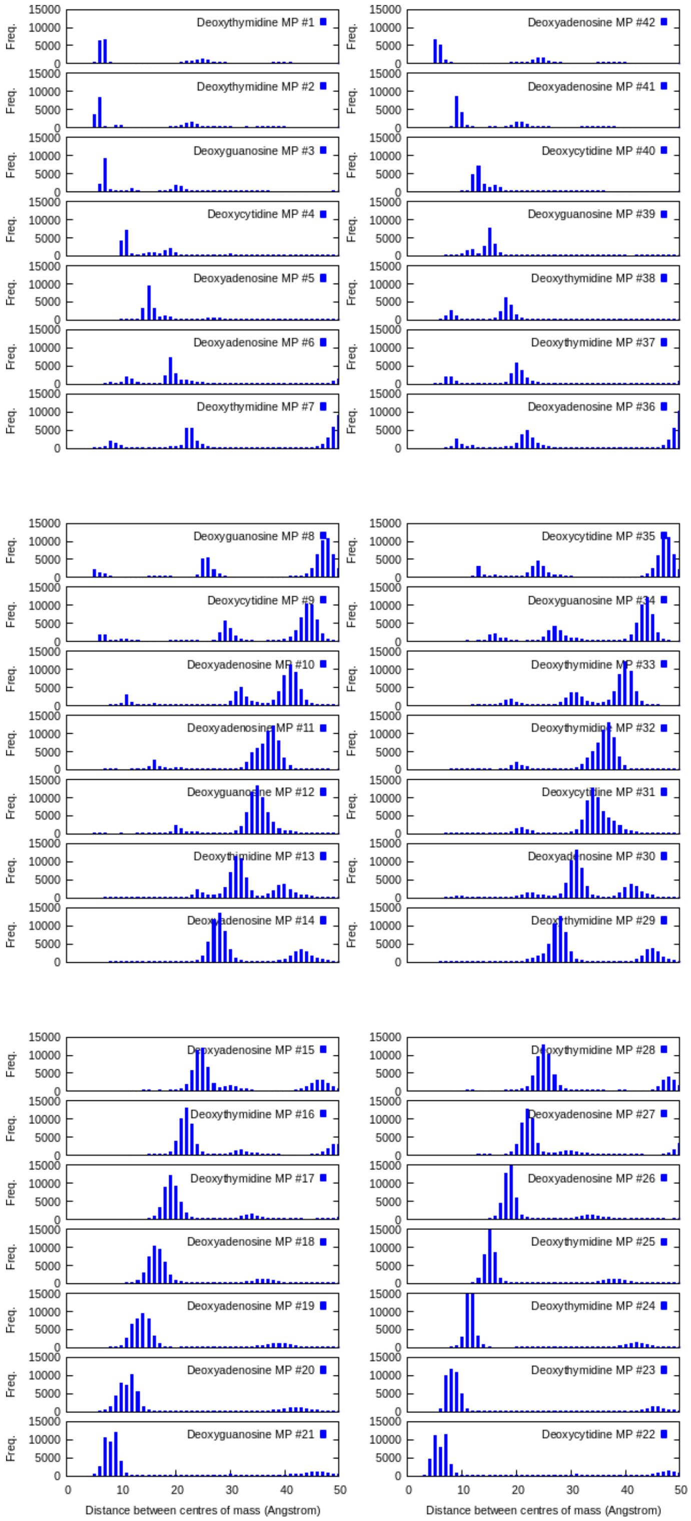

Figure S10. Histogram of the distances between the centre of mass of **4** and the centre of mass of each nucleotide (sequence 5’-TTGCAATGCAAGTAATTAAAG-3’). Data corresponds to the sum of the three MD runs.

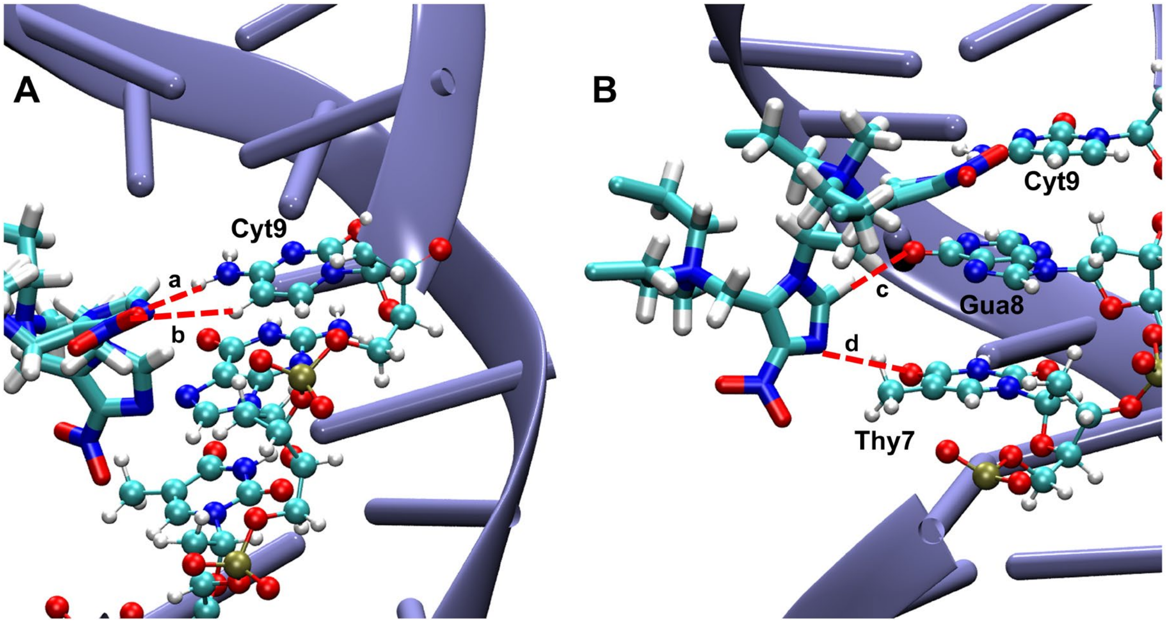

Figure S11. A) An example of hydrogen bonding between **1** and C9 (interactions a and b). B) An example of hydrogen bonding between **1** and G8 (interaction c) and T7 (interaction d).

Validation of a suitable DFT method for QM and QM/MM reactivity

The addition of **4** to the C5 and C6 positions of thymine to *i*) choose a suitable QM model size for the photosensitizer and *ii*) to validate an appropriate DFT method to describe the reactions. Regarding *i*), using initially DFT with implicit solvation, three QM models of different size have been considered (Figure S12). Results are shown in Table S4. The small differences (<2 kcal/mol) between the different models either in the activation energies ($\Delta E^{\ddagger}$) and energy difference between reactants and products (ΔE) clearly indicate that, from an electronic point of view, the reactivity of the photosensitizer can be represented considering only the nitroimidazole ring. In the following QM/MM simulations, the rest of the photosensitizer can thus be safely described using classical force field, only. Regarding *ii*), the functionals B3LYP, ωB97-XD, MN15, and M06-2X were benchmarked to calculate the activation energy and energy difference in the gas phase with respect to DLPNO-CCSD(T). As summarized in Table S5, the M06-2X functional provided the best description of the energy barriers. M06-2X results with a triple-ζ basis set plus polarization and diffusion functions, 6-311+G(2df,2pd), did not show significant deviations with respect to the DLPNO-CCSD(T) reference values and the M06-2X/6-31G* determinations. Therefore, the M06-2X/6-31G* level of theory is subsequently used to model the reactivity of **2** and **4** with the four DNA nucleobases.

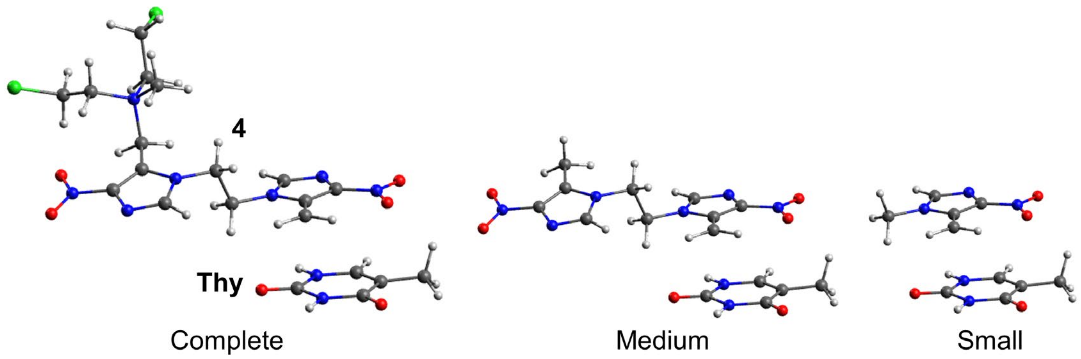

Figure S12. Representation of the three QM models considered in this work.

Table S4. Activation energy ($\Delta E^{\ddagger}$) and energy difference between reactants and products (ΔE) for the addition reaction of **4** to the C5 and C6 positions of thymine in water solution (PCM-B3LYP/6-31G*) considering three different QM models. Energies in kcal/mol.

**QM model**	$\Delta E^{\ddagger}$	ΔE
	C5 addition	
Complete	16.08	9.27
Medium	17.37	9.54
Small	17.75	9.76
	C6 addition	
Complete	15.71	3.25
Medium	17.42	4.80
Small	16.96	4.34

Table S5. Activation energy ($\Delta E^{\ddagger}$) and energy difference between reactants and products (ΔE) for the addition reaction of **4** to the C5 and C6 positions of thymine in the gas phase with different DFT functionals (including empirical dispersion) and a coupled-cluster method. Results are based on the small QM model. Energies in kcal/mol.

**Method**	$\Delta E^{\ddagger}$	ΔE
	C5 addition	
B3LYP/6-31G*	16.24	5.03
ωB97-XD/6-31G*	15.94	-0.80
MN15/6-31G*	18.43	3.78
M06-2X/6-31G*	15.56	-0.29
M06-2X/6-311+G(2df,2pd)[a]	17.31	2.65
M06-2X/6-311+G(2df,2pd)[b]	16.85	2.12
DLPNO-CCSD(T)/6-311+G(2df,2pd)[b]	16.46	0.75
	C6 addition	
B3LYP/6-31G*	14.92	-0.97
ωB97-XD/6-31G*	16.88	-4.95
MN15/6-31G*	18.77	-1.94
M06-2X/6-31G*	17.94	-3.45
M06-2X/6-311+G(2df,2pd)[a]	18.86	-0.90
M06-2X/6-311+G(2df,2pd)[b]	18.25	-1.10
DLPNO-CCSD(T)/6-311+G(2df,2pd)[b]	18.90	-0.56

[a] M06-2X/6-31G* optimized structures.

[b] B3LYP/6-31G* optimized structures.

Table S6. Gibbs energy difference between reactants and products (ΔG) for the addition reaction of **2** to the most relevant positions of the four DNA nucleobases in water solution with the PCM-M06-2X/6-31G* method including basis set superposition error corrections. Results are based on the small QM model. All ΔG values are in kcal/mol and are relative to **2** and the nucleobase at an infinite distance.

**Reaction channel**	**ΔG**
Thymine	
N1	1.13
O2	-15.20
N3	1.09
O4	-12.17
C5	-9.76
C6	Unstable
Cytosine	
N1	-3.20
O2	-30.61
N3	-37.51
N4	-16.12
C5	-17.77
C6	Unstable
Guanine	
N1	4.74
N2	-15.23
N3	-25.77
C4	5.97
C2	25.45
C5	-21.22
O6	-24.50
N7	-39.43
C8	-24.46
Adenine	
N1	-36.38
C2	10.88
N3	-36.47
C4	12.62
C5	-5.52
N6	-23.05
N7	-30.24
C8	-10.49

Table S7. Activation Gibbs energy ($\Delta G^{\ddagger}$) and Gibbs energy difference between reactants and products (ΔG) for the addition reaction of **4** to the most relevant positions of the four DNA nucleobases in water solution with the PCM-M06-2X/6-31G* method. Results are based on the small QM model. Energies in kcal/mol.

**Reaction channel**	$\Delta G^{\ddagger}$	ΔG
Thymine		
C5	16.90	4.10
C6	17.54	-1.00
H abstraction from the methyl group	17.83	0.35
Cytosine		
C5	17.15	3.29
C6	20.48	3.36
Guanine		
C2	22.59	11.58
C4	24.81	13.95
C5	20.33	16.19
C8	13.67	-1.33
Adenine		
C2	21.61	8.12
C4	27.79	20.94
C5	24.55	17.32
C8	14.91	-0.62

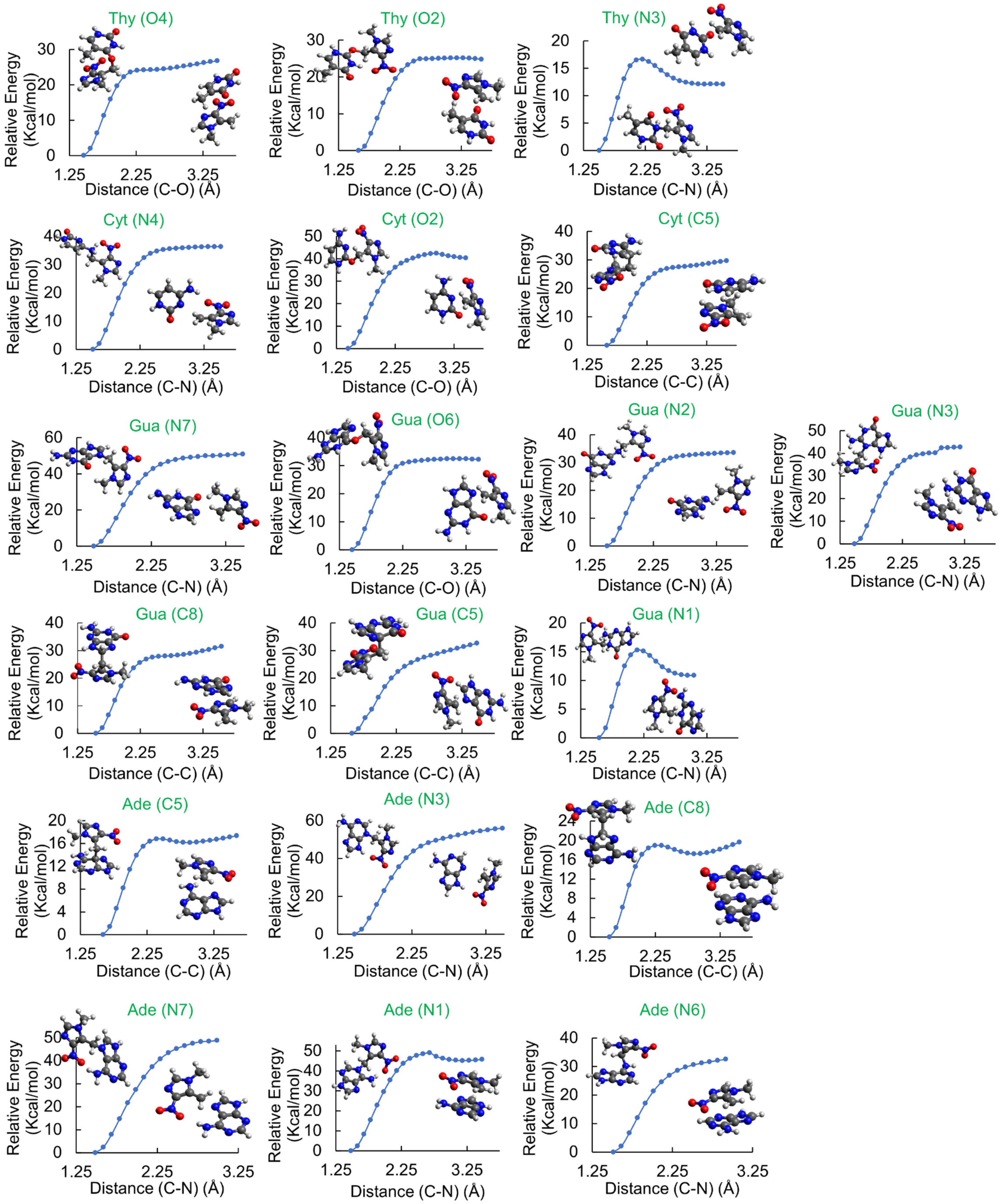

Figure S13. Relevant relaxed scan for the addition of a model of **2** toward different positions of the DNA nucleobases.

Table S8. Time until the reaction occurs (in ps and in bold) between **2** and the N7, O6, and N2 positions of G8 and G34 as obtained with several QM/MM runs. t = 0 corresponds to the first frame of the QM/MM run. NR denotes no reaction observed in the 10 ps simulation. The initial distance (t = 0 ps) between the atoms involved in the reactions (*i.e.* C-N or C-O) is shown in italics and within parenthesis. The average reaction time and the total number of reactions (within parenthesis) are listed in the last row.

	**G8 (major groove)**		**G34 (major groove)**		**G34 (minor groove)**
**Run**	**N7**	**O6**	**N7**	**O6**	**N2**
**1**	**9.25**<br>(*3.61*)	(*5.21*)	**4.30**<br>(*2.94*)	(*3.99*)	NR<br>(*3.16*)
**2**	(*3.95*)	**1.45**<br>(*3.06*)	**6.70**<br>(*3.10*)	(*3.99*)	**7.95**<br>(*3.53*)
**3**	NR<br>(*3.77*)	NR<br>(*3.94*)	NR<br>(*3.48*)	NR<br>(*4.23*)	NR<br>(*2.94*)
**4**	NR<br>(*5.95*)	NR<br>(*3.72*)	NR<br>(*3.93*)	NR<br>(*5.96*)	NR<br>(*2.96*)
**5**	(*4.01*)	**0.25**<br>(*2.66*)	**3.30**<br>(*3.20*)	(*3.67*)	NR<br>(*3.17*)
**6**	(*4.21*)	**0.70**<br>(*2.94*)	NR<br>(*3.05*)	**0.85**<br>(*3.69*)	NR<br>(*3.03*)
**7**	(*4.17*)	**0.45**<br>(*2.96*)	NR<br>(*5.88*)	NR<br>(*8.06*)	NR<br>(*3.34*)
**8**	(*4.97*)	**0.85**<br>(*2.93*)	NR<br>(*3.36*)	0.95<br>(*3.01*)	NR<br>(*3.17*)
**9**			NR<br>(*4.56*)	0.95<br>(*3.81*)	
**Avg.**	**9.25**<br>(1/7)	**0.74**<br>(5/7)	**4.77**<br>(3/9)	**0.92**<br>(3/9)	**7.95**<br>(1/8)

Table S9. Time until the reaction occurs (in ps and in bold) between **2** and the N4 and O2 positions of C9 and C35 as obtained with several QM/MM runs. t = 0 corresponds to the first frame of the QM/MM run. Same format as in Table S6.

	**C9**	**C35**	**C35**
**Run**	**N4 (major groove)**	**N4 (major groove)**	**O2 (minor groove)**
**1**	NR<br>(*3.77*)	**0.25**<br>(*3.10*)	**9.15**<br>(*4.22*)
**2**	NR<br>(*3.09*)	NR<br>(*4.85*)	NR<br>(*3.94*)
**3**	NR<br>(*5.79*)	NR<br>(*3.84*)	NR<br>(*4.19*)
**4**	NR<br>(*4.65*)	NR<br>(*3.45*)	NR<br>(*4.28*)
**5**	**5.25**<br>(*3.46*)	NR<br>(*3.36*)	NR<br>(*3.87*)
**6**	**5.40**<br>(*3.72*)	NR<br>(*3.54*)	NR<br>(*3.99*)
**7**	NR<br>(*4.45*)	NR<br>(*3.61*)	NR<br>(*4.47*)
**8**	NR<br>(*3.47*)		
**Avg.**	**5.32**<br>(2/8)	**0.25**<br>(1/7)	**9.15**<br>(1/7)

Table S10. Time until the reaction occurs (in ps and in bold) between **2** and the N3 position of A10 and A18 as obtained with several QM/MM runs. t = 0 corresponds to the first frame of the QM/MM run. Same format as in Table S6.

	**A10**	**A18**
**Run**	**N3<br>(minor groove)**	**N3<br>(minor groove)**
**1**	NR<br>*(3.13)*	**9.20**<br>*(3.39)*
**2**	**0.85**<br>*(3.15)*	**2.60**<br>*(3.08)*
**3**	**5.00**<br>*(2.99)*	**8.50**<br>*(3.29)*
**4**	**8.45**<br>*(3.14)*	**9.25**<br>*(3.44)*
**5**	**0.8**<br>*(2.94)*	NR<br>*(3.72)*
**6**	NR<br>*(2.87)*	NR<br>*(2.99)*
**7**	**0.6**<br>*(2.86)*	NR<br>*(2.99)*
**8**	NR<br>*(2.97)*	**6.90**<br>*(3.06)*
**Avg.**	**3.14**<br>(5/8)	**7.29**<br>(5/8)

Table S11. Time until the reaction occurs (in ps and in bold) between **2** and the C5, O4 and O2 positions of T13 and T25 as obtained with several QM/MM runs. t = 0 corresponds to the first frame of the QM/MM run. Same format as in Table S6.

	**T25<br>(major groove)**		**T25<br>(minor<br>groove)**	**T13<br>(major groove)**	
**Run**	**C5**	**O4**	**O2**	**C5**	**O4**
**1**	NR<br>*(4.48)*	NR<br>*(5.87)*	NR<br>*(3.79)*	NR<br>*(5.44)*	NR<br>*(3.30)*
**2**	NR<br>*(4.29)*	NR<br>*(4.13)*	NR<br>*(3.47)*	NR<br>*(6.24)*	NR<br>*(6.48)*
**3**	NR<br>*(4.97)*	NR<br>*(5.94)*	NR<br>*(3.19)*	NR<br>*(5.79)*	NR<br>*(7.09)*
**4**	NR<br>*(4.20)*	NR<br>*(5.74)*	**0.55**<br>*(3.09)*	NR<br>(5.81)	NR<br>*(3.67)*
**5**	NR<br>*(5.15)*	NR<br>*(6.26)*	NR<br>*(2.83)*	NR<br>(6.00)	NR<br>*(7.03)*
**6**	NR<br>*(4.98)*	NR<br>*(6.98)*	**2.55**<br>*(2.97)*	NR<br>(5.18)	NR<br>(6.17)
**7**	NR<br>*(4.99)*	NR<br>*(5.99)*	**8.20**<br>*(2.74)*	*(4.53)*	**0.85**<br>(4.62)
**8**				NR<br>(4.11)	NR<br>(4.93)
**9**				NR<br>(5.91)	NR<br>(4.84)
**Avg.**	**NR**<br>(0/7)	**NR**<br>(0/7)	**3.77**<br>(3/7)	**NR**<br>(0/9)	**0.85**<br>(1/9)

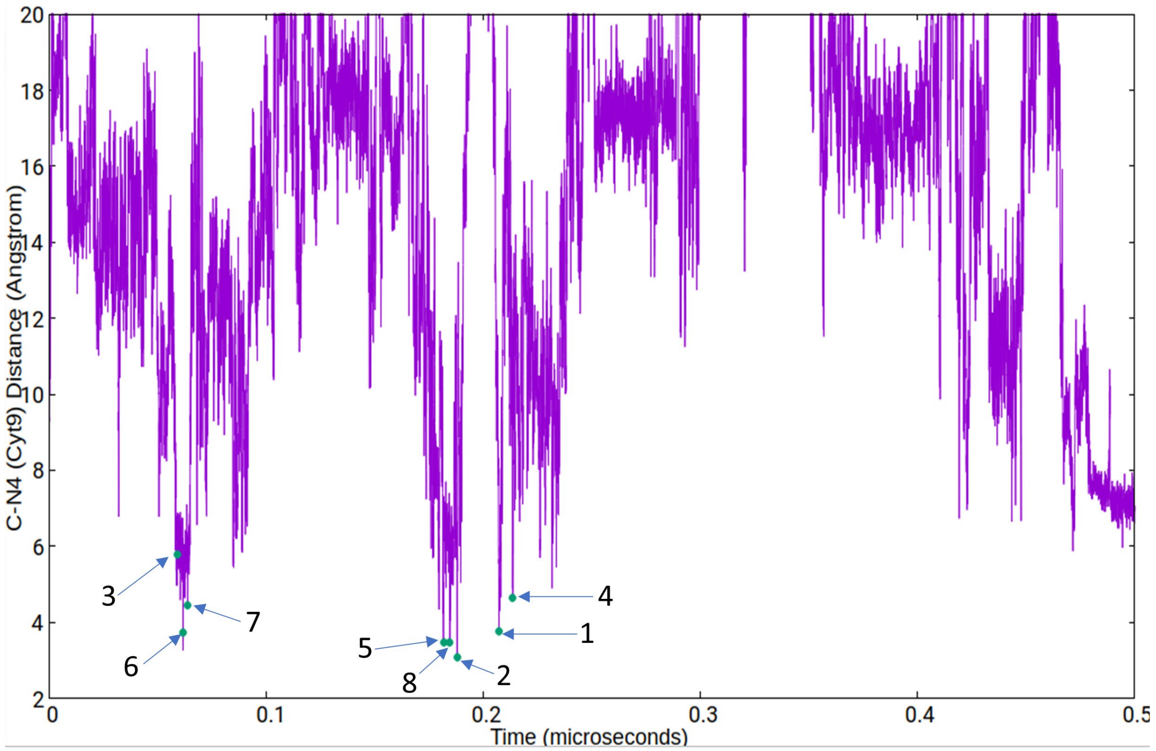

Figure S14. C-N4 (Cyt9) distance evolution versus time of the 1-microsecond simulation. Dots and arrows with numbers refer to the QM/MM run number in Tables S6-S9.

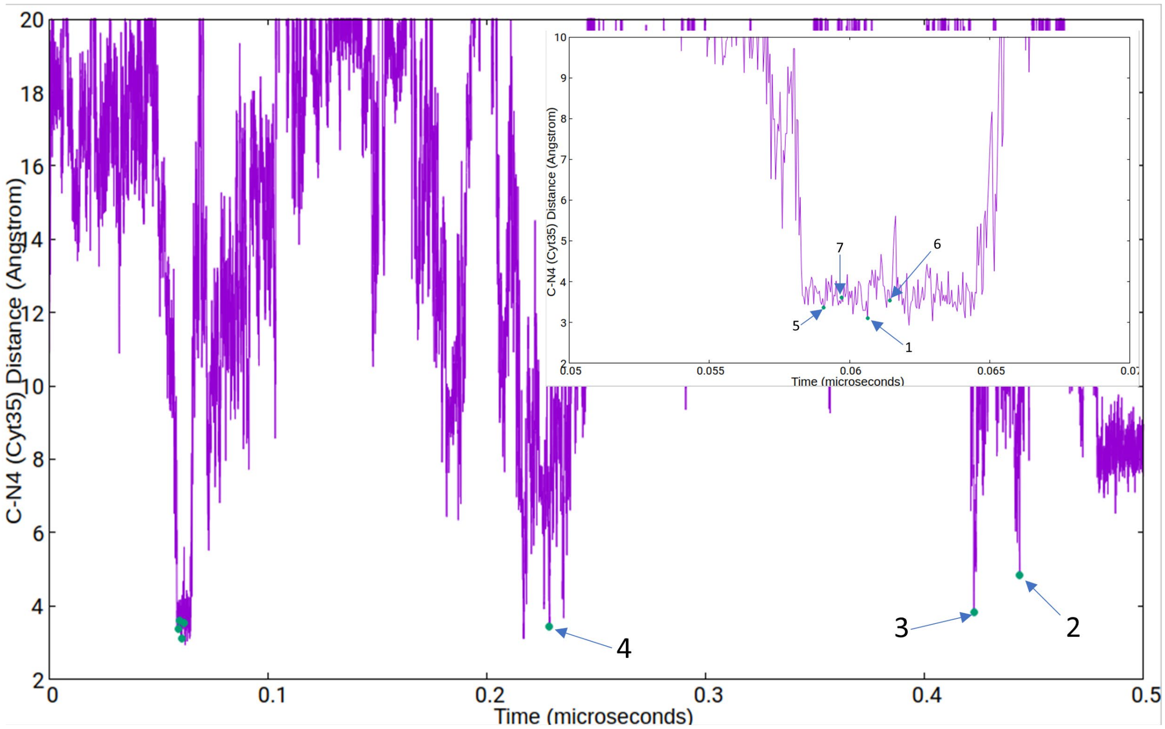

Figure S15. C-N4 (Cyt35) distance evolution versus time of the 1-microsecond simulation. Dots and arrows with numbers refer to the QM/MM run number in Tables S6-S9.

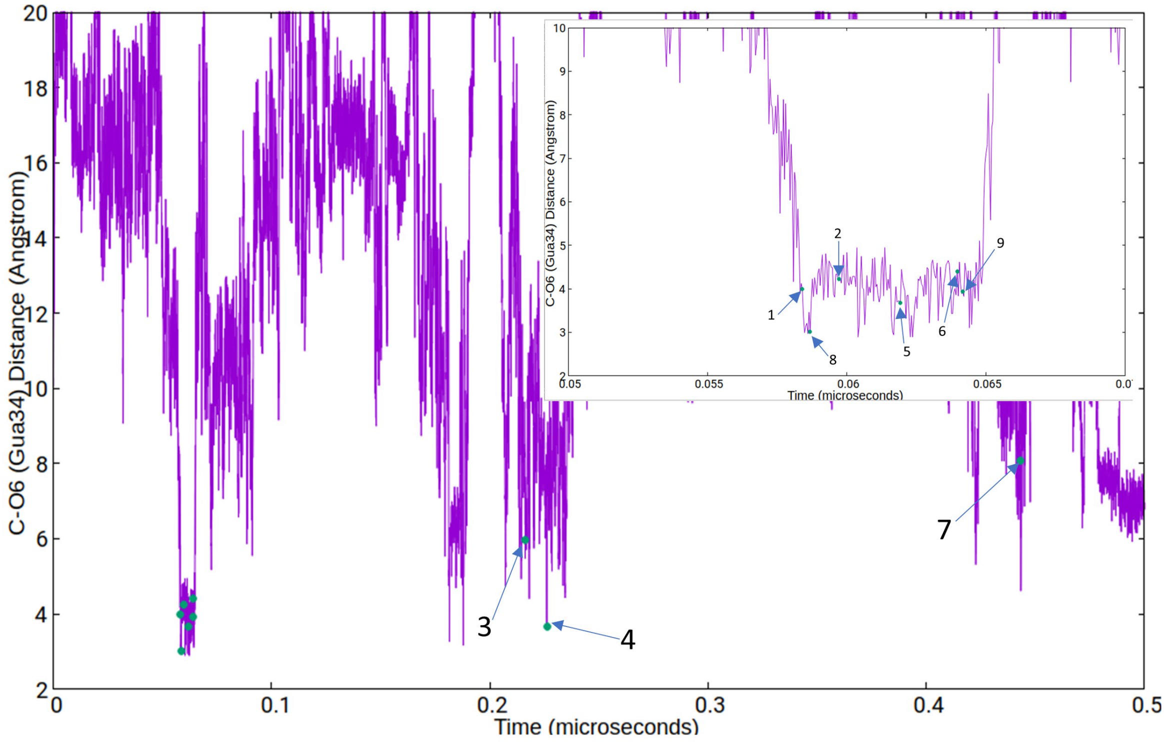

Figure S16. C-O6 (Gua34) distance evolution versus time of the 1-microsecond simulation. Dots and arrows with numbers refer to the QM/MM run number in Tables S6-S9.

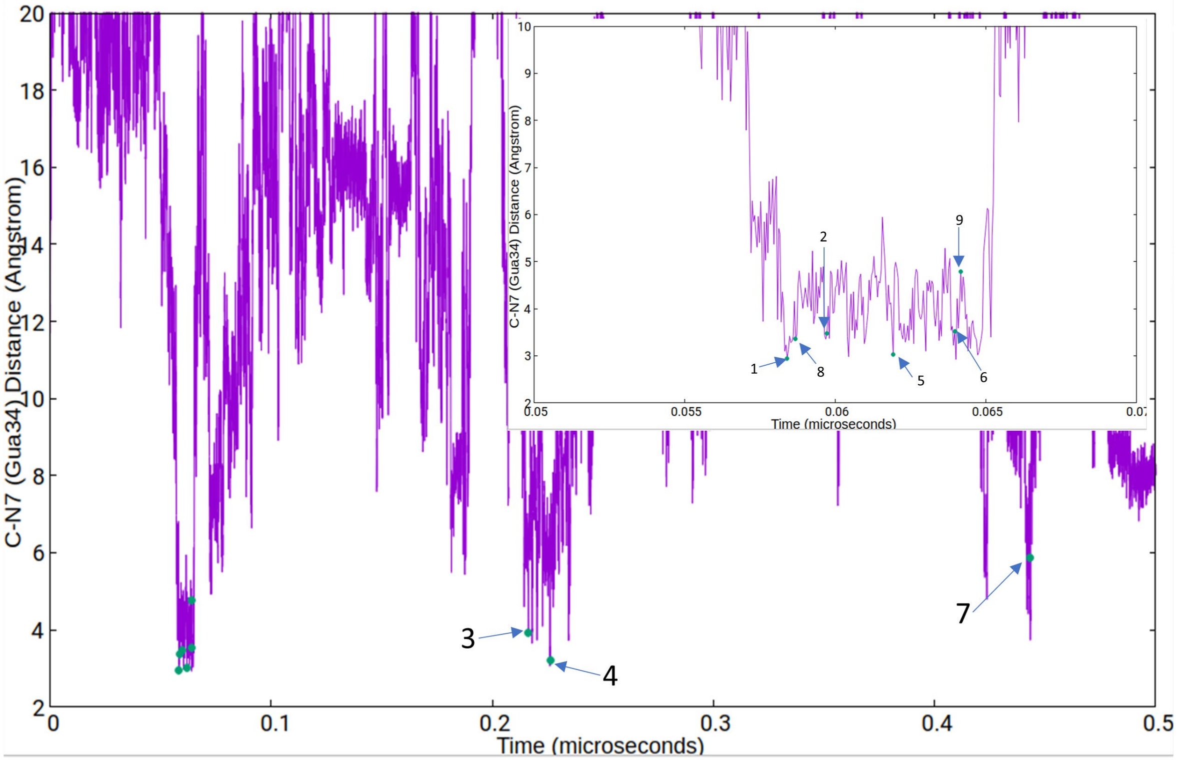

Figure S17. C-N7 (Gua34) distance evolution versus time of the 1-microsecond simulation. Dots and arrows with numbers refer to the QM/MM run number in Tables S6-S9.

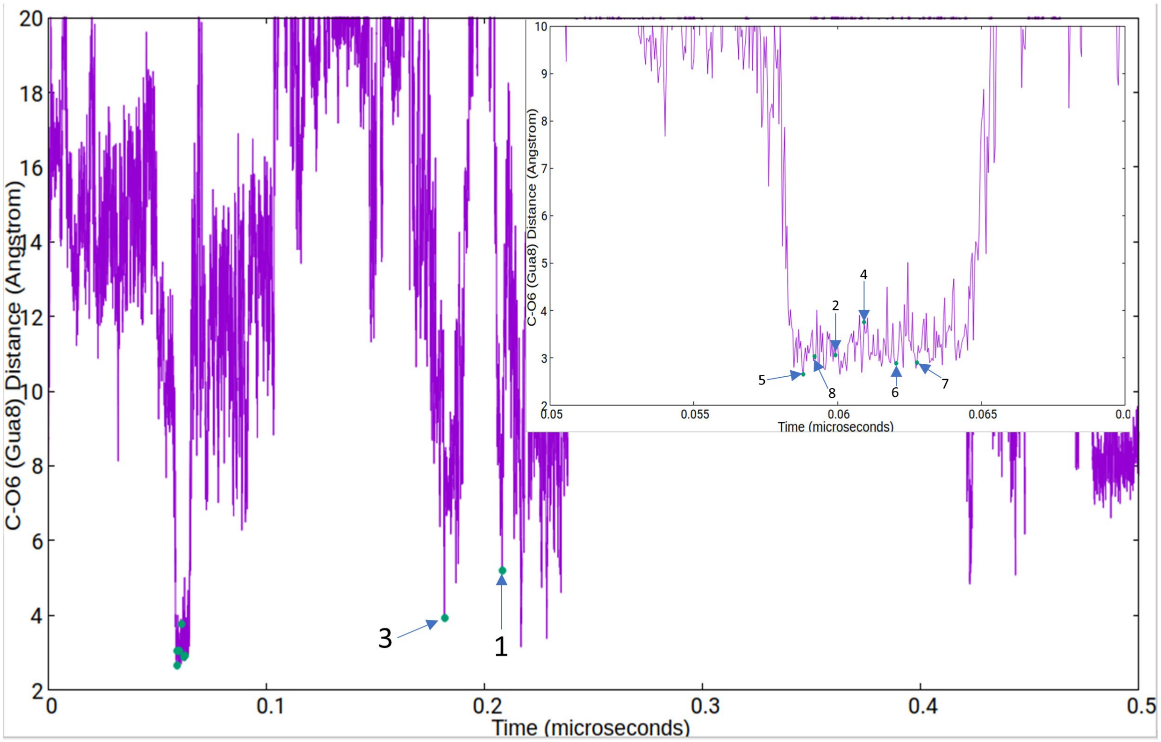

Figure S18. C-O6 (Gua8) distance evolution versus time of the 1-microsecond simulation. Dots and arrows with numbers refer to the QM/MM run number in Tables S6-S9.

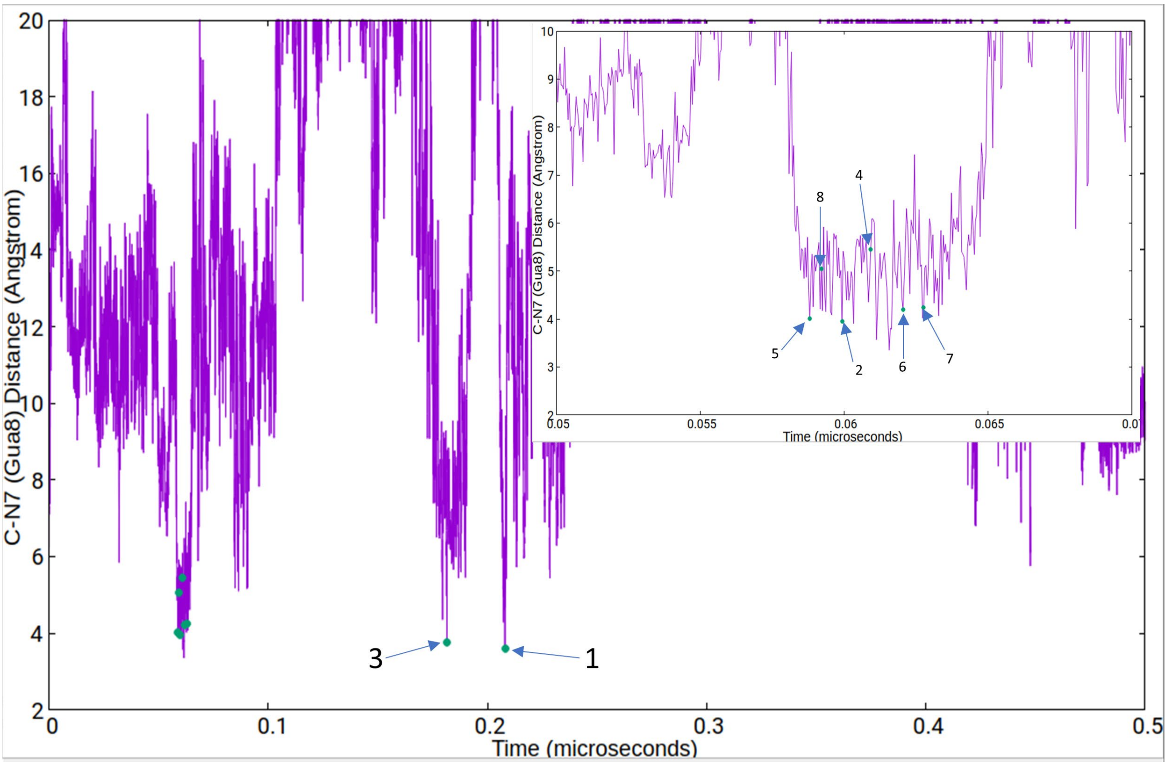

Figure S19. C-N7 (Gua8) distance evolution versus time of the 1-microsecond simulation. Dots and arrows with numbers refer to the QM/MM run number in Tables S6-S9.

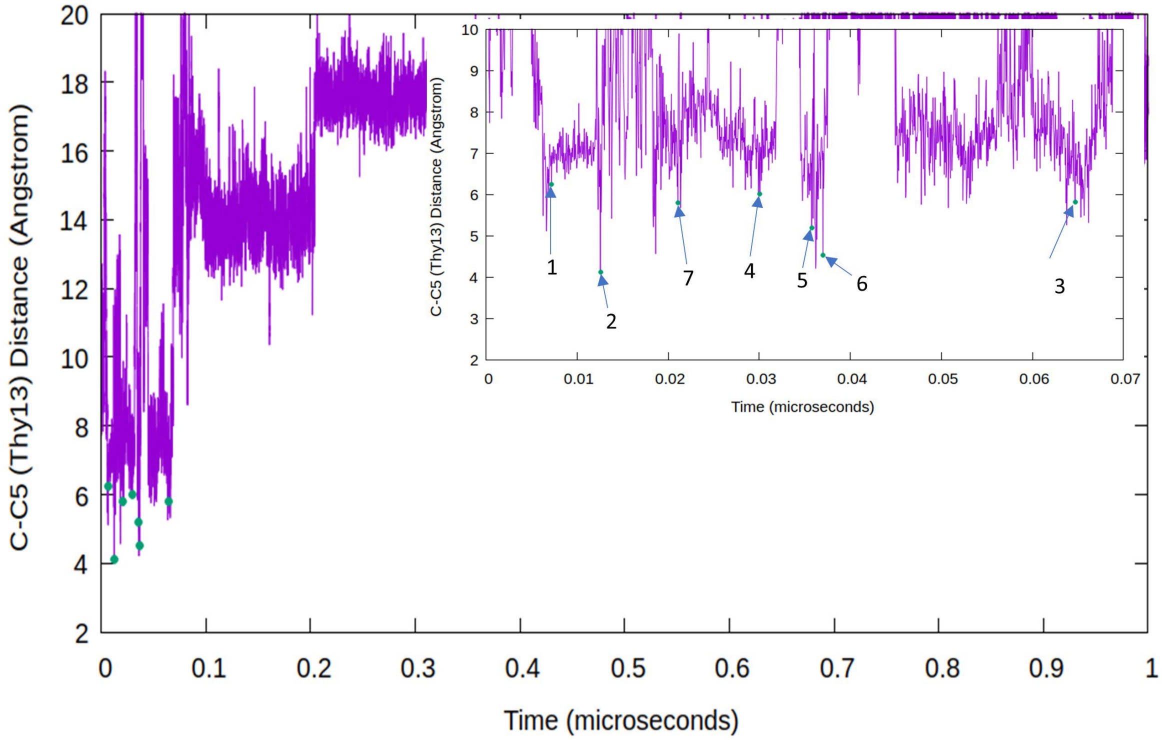

Figure S20. C-C5 (Thy13) distance evolution versus time of the 1-microsecond simulation. Dots and arrows with numbers refer to the QM/MM run number in Tables S6-S9.

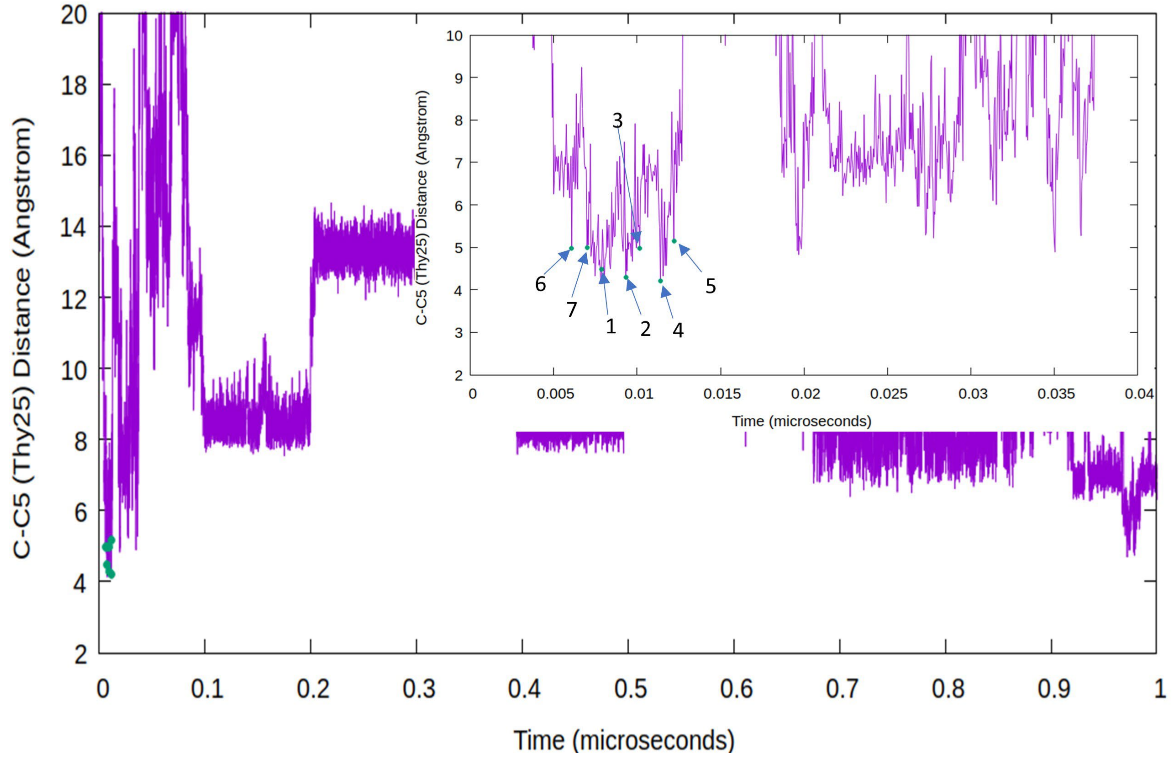

Figure S21. C-C5 (Thy25) distance evolution versus time of the 1-microsecond simulation. Dots and arrows with numbers refer to the QM/MM run number in Tables S6-S9.

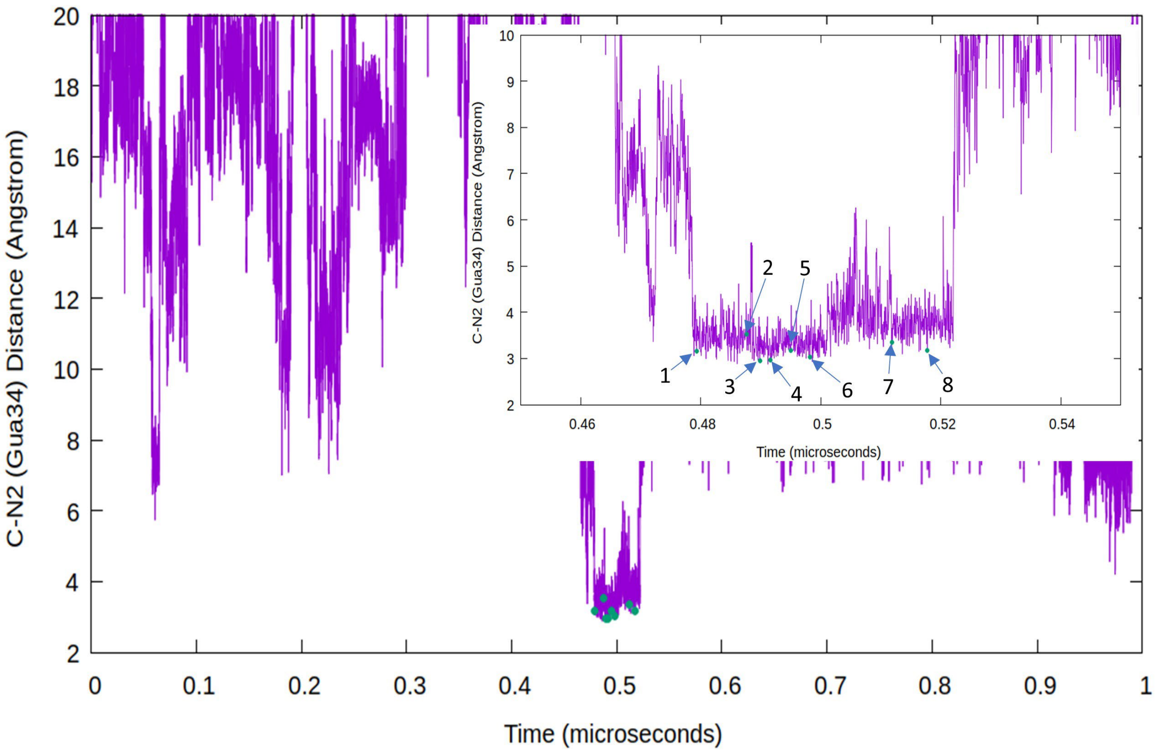

Figure S22. C-N2 (Gua34) distance evolution versus time of the 1-microsecond simulation. Dots and arrows with numbers refer to the QM/MM run number in Tables S6-S9.

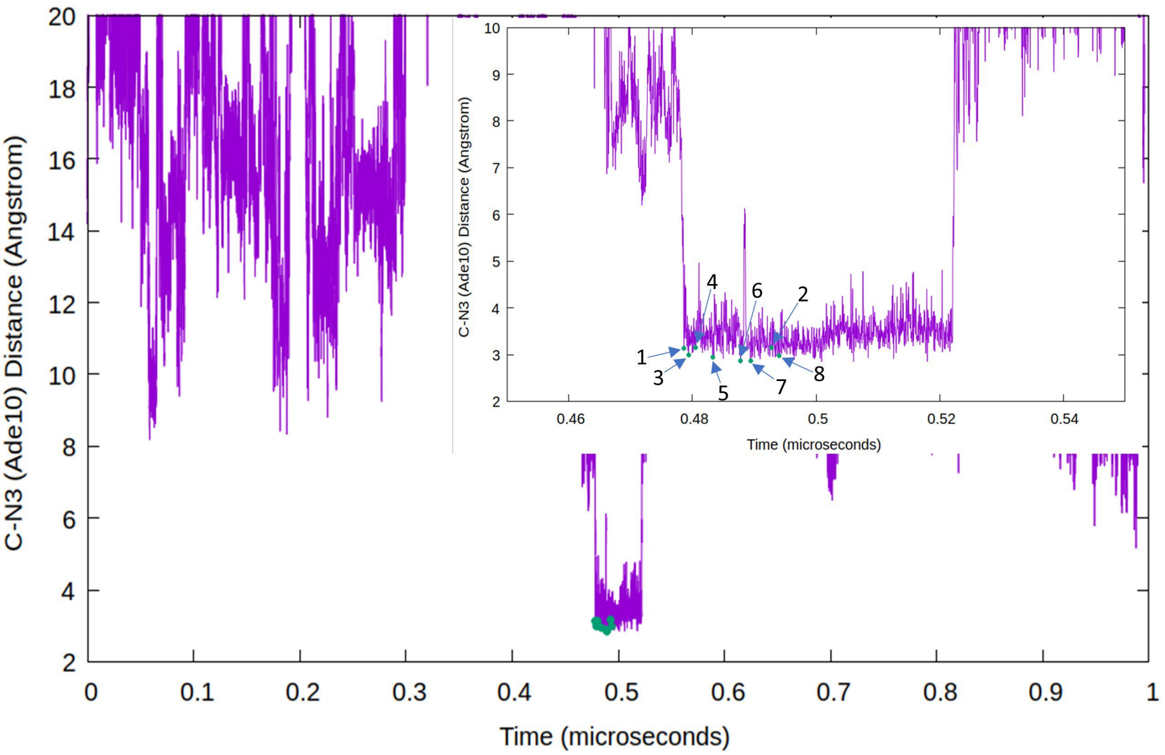

Figure S23. C-N3 (Ade34) distance evolution versus time of the 1-microsecond simulation. Dots and arrows with numbers refer to the QM/MM run number in Tables S6-S9.

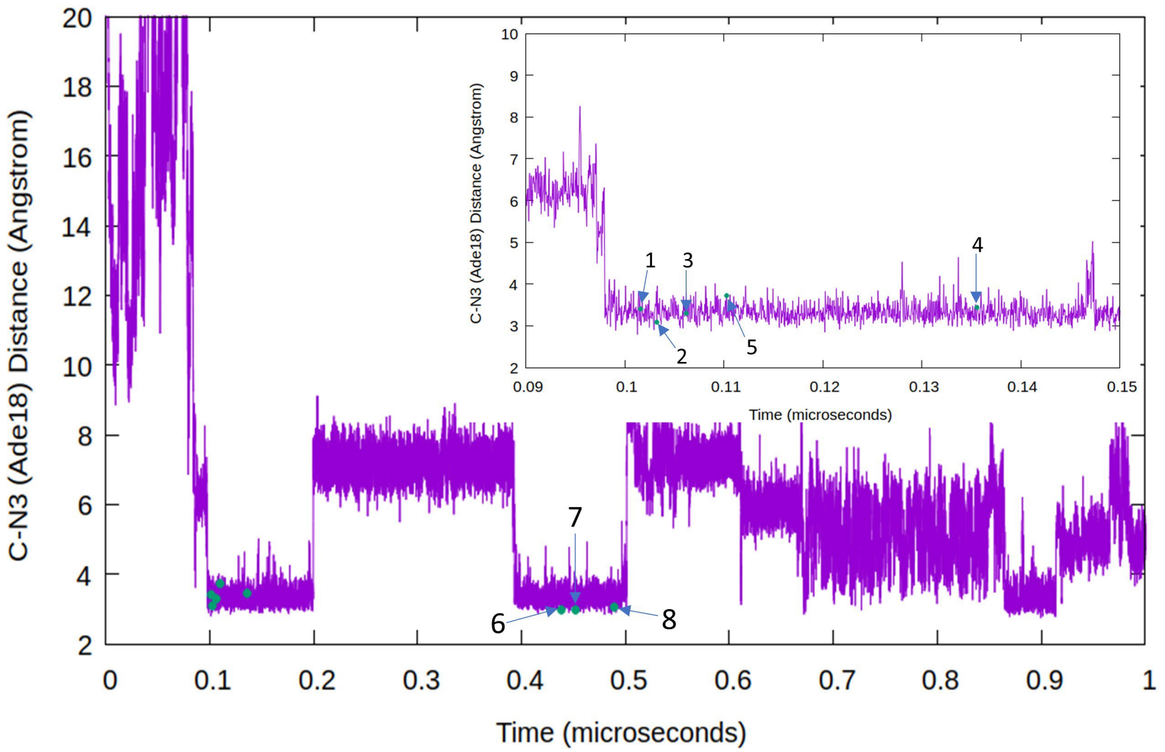

Figure S24. C-N3 (Ade18) distance evolution versus time of the 1-microsecond simulation. Dots and arrows with numbers refer to the QM/MM run number in Tables S6-S9.

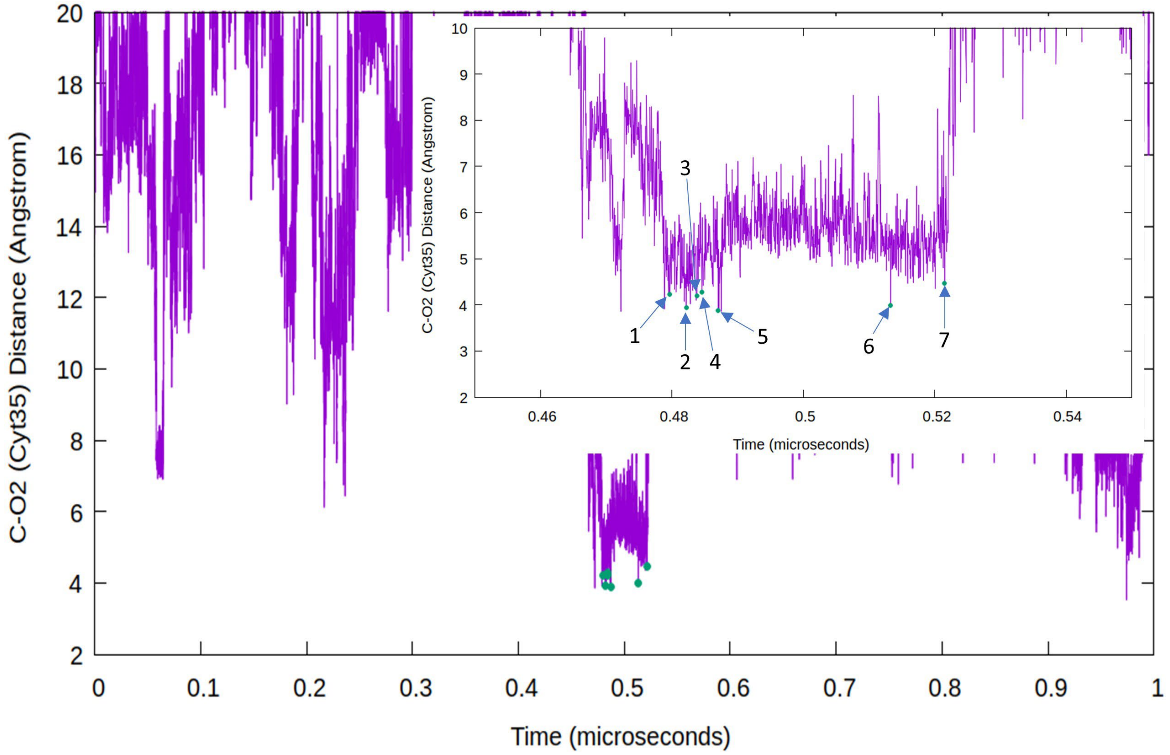

Figure S25. C-O2 (Cyt35) distance evolution versus time of the 1-microsecond simulation. Dots and arrows with numbers refer to the QM/MM run number in Tables S6-S9.

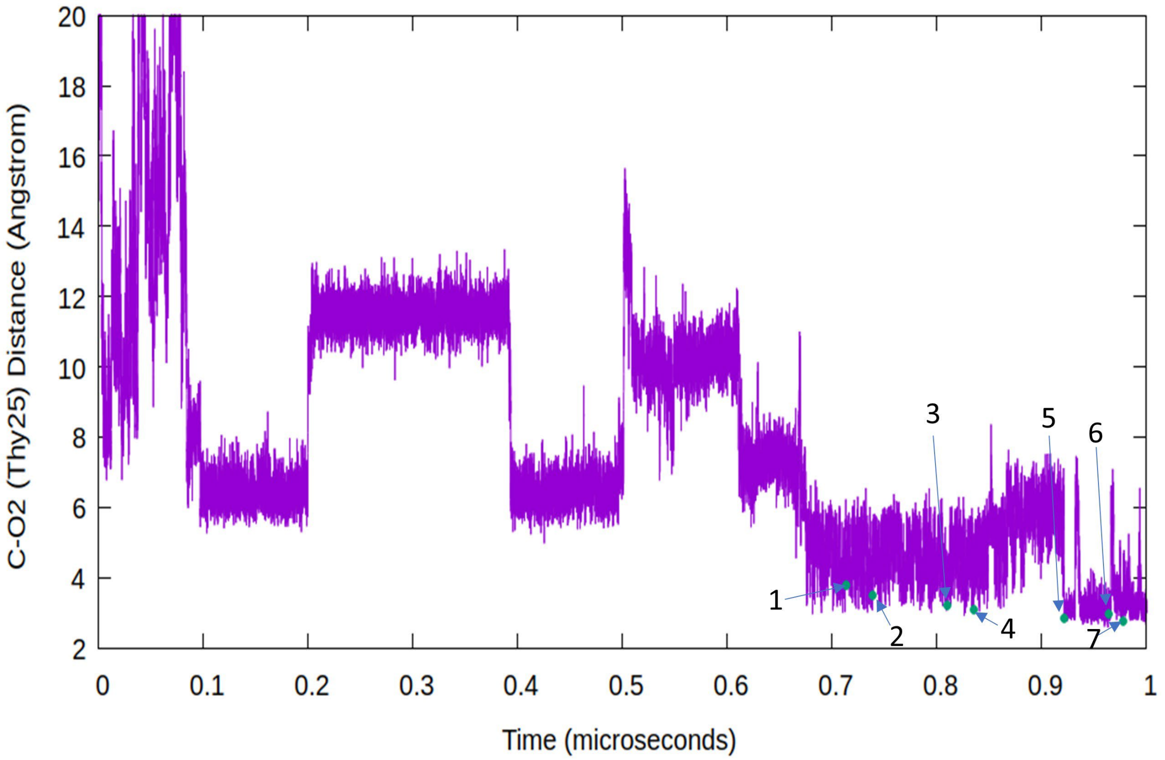

Figure S26. C-O2 (Thy25) distance evolution versus time of the 1-microsecond simulation. Dots and arrows with numbers refer to the QM/MM run number in Tables S6-S9.

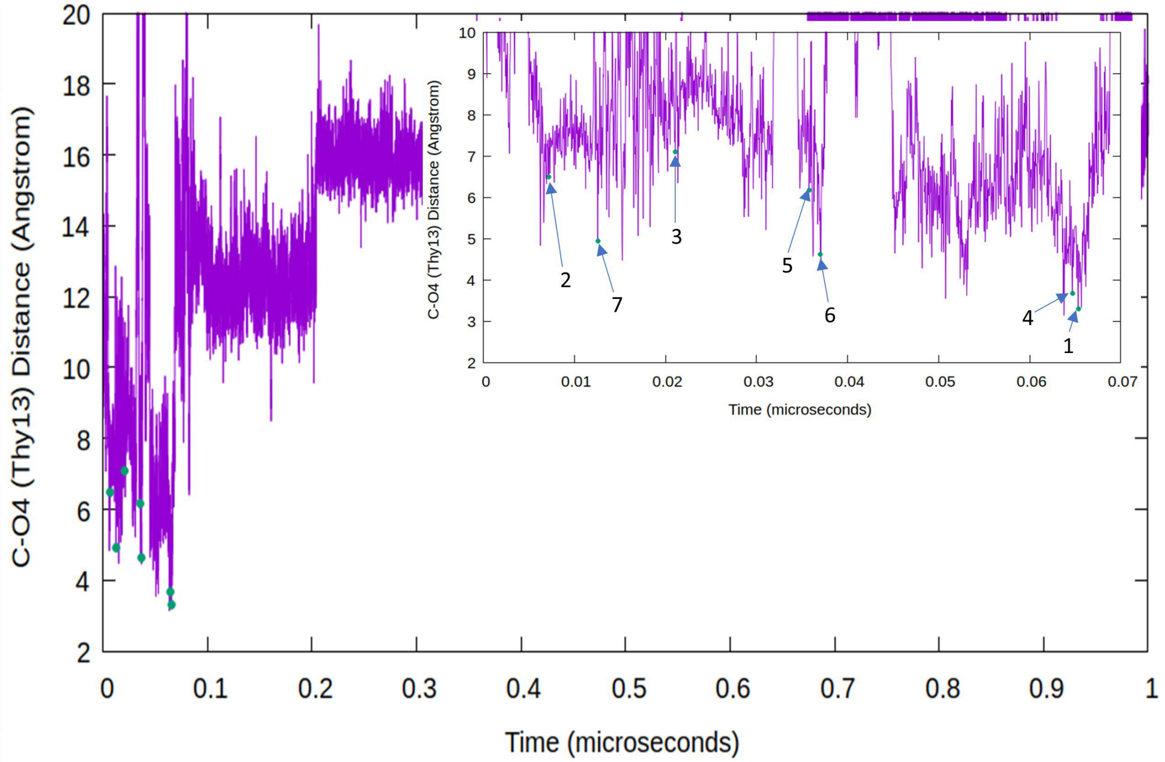

Figure S27. C-O4 (Thy13) distance evolution versus time of the 1-microsecond simulation. Dots and arrows with numbers refer to the QM/MM run number in Tables S6-S9.

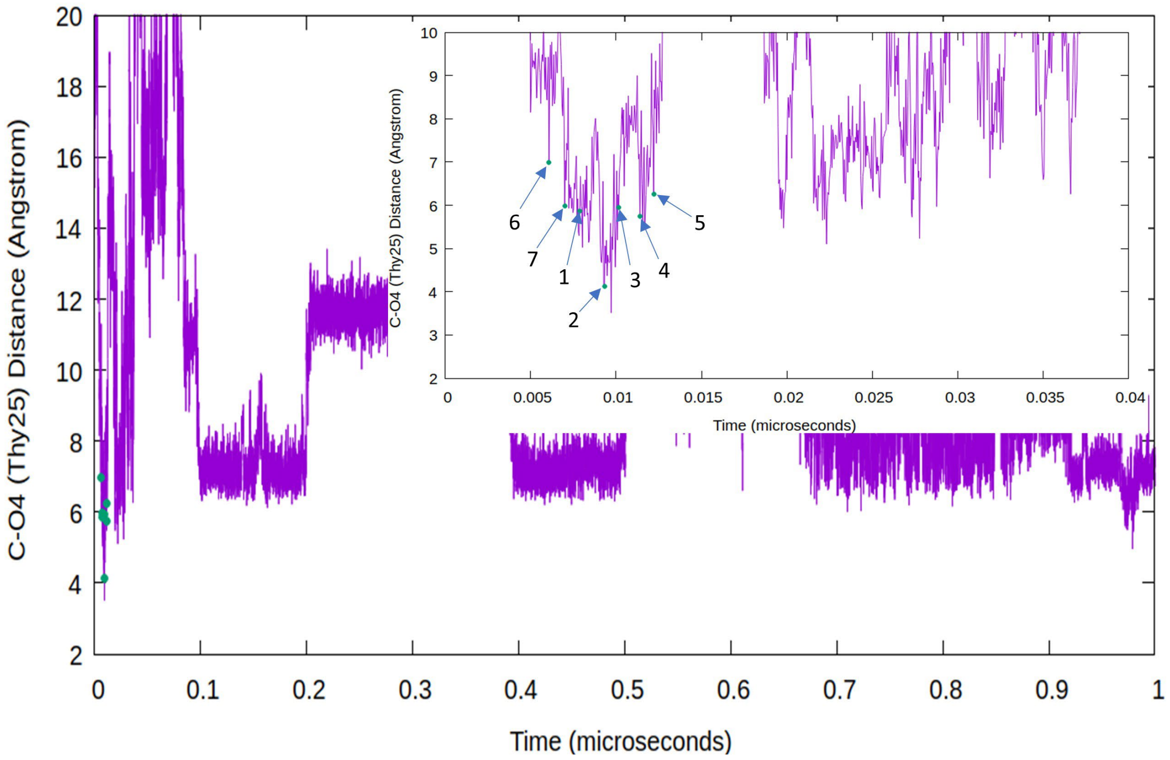

Figure S28. C-O4 (Thy25) distance evolution versus time of the 1-microsecond simulation. Dots and arrows with numbers refer to the QM/MM run number in Tables S8-S11.

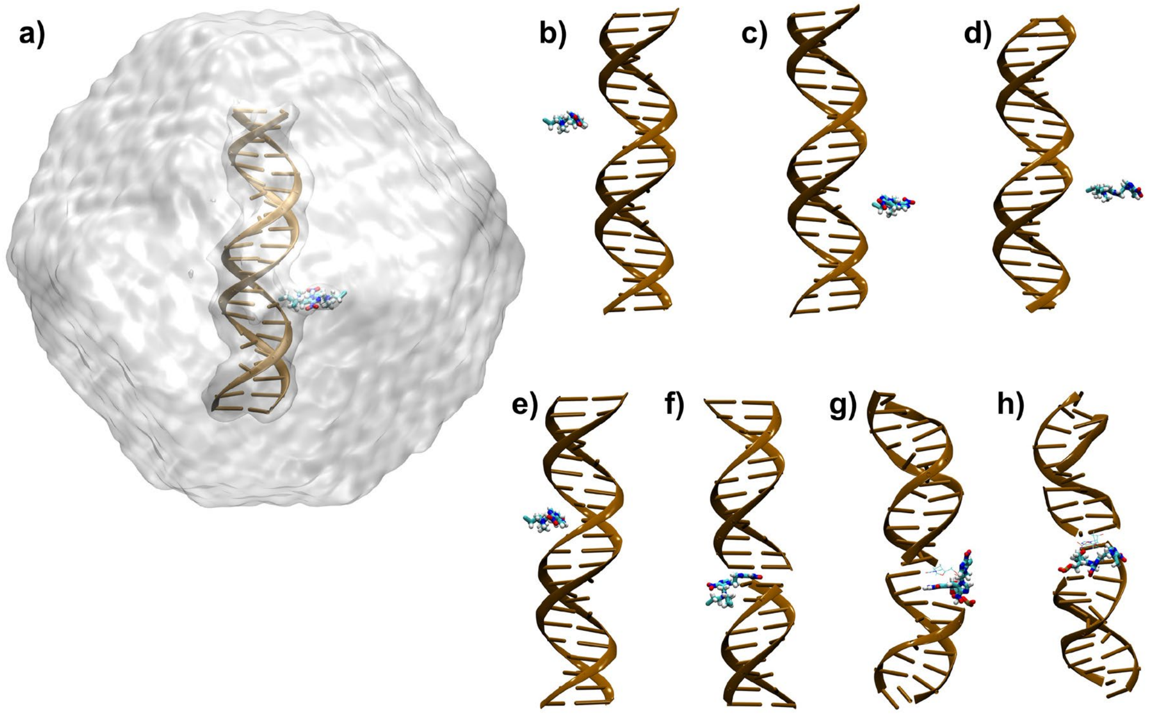

Figure S29. 3D representations of the starting DNA + photosensitizer structures simulated with classical MD prior minimization and heating. a) **1** + DNA (also shows the truncated octahedral water box), b) **2** + DNA (run #1), c) **2** + DNA (run #2), d) **4** + DNA (run #1), e) **4** + DNA (run #2), f) **4** + DNA (run #3, staring from an intercalated configuration), g) **6** on G8, and h) **11** on A10. In g) and h), the adjacent stacked nucleobase to the DNA lesion is represented with wires.

Atom types and point charges for the force fields obtained through the RESP procedure

Species **1** (GAFF)

Atom #	Label	x	y	z	Atom type	Point charge
1	C1	-2.925	2.487	-0.433	cc	0.578016
2	N1	-1.939	3.215	0.136	nc	-0.515123
3	C2	-2.69	1.118	-0.375	cd	-0.339702
4	C3	-1.067	2.328	0.541	cd	0.18758
5	N2	-1.444	1.036	0.259	na	-0.132501
6	C4	-0.526	-0.099	0.375	c3	0.094232
7	C5	0.571	-0.06	-0.723	c3	0.094232
8	N3	1.488	-1.196	-0.609	na	-0.132501
9	C6	2.731	-1.288	0.03	cc	-0.339702
10	C7	1.116	-2.48	-0.927	cc	0.18758
11	C8	2.969	-2.658	0.054	cd	0.578016
12	N4	1.989	-3.374	-0.539	nd	-0.515123
13	N5	-4.047	3.166	-1.079	no	0.417798
14	C9	-3.505	-0.025	-0.873	c3	-0.051012
15	N6	-4.569	-0.643	0.102	n4	0.342433
16	N7	4.078	-3.349	0.701	no	0.417798
17	C10	3.513	-0.121	0.533	c3	-0.051012
18	N8	4.587	0.491	-0.431	n4	0.342433
19	C11	5.889	-0.335	-0.452	c3	-0.032975
20	C12	4.838	1.919	0.072	c3	-0.032975
21	C13	4.063	0.533	-1.842	c3	-0.128699
22	C14	6.739	-0.295	0.818	c3	-0.49249
23	Cl1	8.197	-1.284	0.475	cl	-0.021296
24	C15	5.917	2.715	-0.665	c3	-0.49249
25	Cl2	5.981	4.329	0.119	cl	-0.021296
26	C16	-5.374	0.489	0.761	c3	-0.032975
27	C17	-5.45	-1.526	-0.807	c3	-0.032975
28	C18	-3.869	-1.455	1.154	c3	-0.128699
29	C19	-6.607	0.079	1.568	c3	-0.49249
30	Cl3	-7.195	1.568	2.381	cl	-0.021296
31	C20	-6.2	-2.684	-0.14	c3	-0.49249
32	Cl4	-7.126	-3.5	-1.443	cl	-0.021296
33	H1	-0.144	2.555	1.06	h5	0.176177
34	H2	-0.068	-0.073	1.369	h1	0.069636
35	H3	-1.089	-1.031	0.306	h1	0.069636
36	H4	0.113	-0.085	-1.717	h1	0.069636
37	H5	1.133	0.874	-0.655	h1	0.069636

38	H6	0.196	-2.696	-1.456	h5	0.176177
39	H7	-4.671	1.007	1.416	hx	0.107433
40	O1	-4.063	4.377	-1.061	o	-0.289305
41	H8	-4.08	0.327	-1.729	hx	0.09834
42	H9	-2.89	-0.876	-1.175	hx	0.09834
43	O2	4.113	-4.559	0.64	o	-0.289305
44	O3	4.892	-2.62	1.29	o	-0.289305
45	H10	2.853	0.715	0.771	hx	0.09834
46	H11	4.053	-0.408	1.433	hx	0.09834
47	H12	5.597	-1.361	-0.669	hx	0.107433
48	H13	6.467	0.046	-1.295	hx	0.107433
49	H14	3.885	2.446	-0.014	hx	0.107433
50	H15	5.088	1.84	1.132	hx	0.107433
51	H16	3.147	1.127	-1.869	hx	0.095717
52	H17	4.809	0.991	-2.49	hx	0.095717
53	H18	3.87	-0.484	-2.181	hx	0.095717
54	H19	7.084	0.707	1.073	h1	0.247525
55	H20	6.239	-0.745	1.675	h1	0.247525
56	H21	6.91	2.272	-0.584	h1	0.247525
57	H22	5.68	2.876	-1.718	h1	0.247525
58	O4	-4.891	2.431	-1.622	o	-0.289305
59	H23	-5.663	1.165	-0.044	hx	0.107433
60	H24	-6.147	-0.846	-1.303	hx	0.107433
61	H25	-4.787	-1.944	-1.567	hx	0.107433
62	H26	-3.185	-0.803	1.697	hx	0.095717
63	H27	-4.596	-1.865	1.854	hx	0.095717
64	H28	-3.326	-2.275	0.679	hx	0.095717
65	H29	-7.42	-0.278	0.935	h1	0.247525
66	H30	-6.399	-0.654	2.349	h1	0.247525
67	H31	-5.524	-3.425	0.289	h1	0.247525
68	H32	-6.913	-2.367	0.619	h1	0.247525

Species **2** (GAFF)

Atom #	Label	x	y	z	Atom type	Point charge
1	C1	5.752	0.63	-0.319	cc	0.609164
2	N1	5.158	1.456	-1.122	nd	-0.495937
3	C2	4.904	-0.491	0.06	cc	-0.329909
4	C3	3.916	0.933	-1.308	cd	0.436052
5	N2	3.696	-0.196	-0.647	na	0.026369
6	C4	2.449	-0.987	-0.672	c3	-0.222031
7	C5	1.485	-0.632	0.492	c3	0.058099
8	N3	0.325	-1.521	0.489	na	-0.027925

9	C6	-0.953	-1.341	-0.066	cc	-0.312234
10	C7	0.415	-2.851	0.833	cc	0.144482
11	C8	-1.491	-2.618	-0.022	cd	0.519833
12	N4	-0.662	-3.522	0.536	nd	-0.476637
13	N5	7.159	0.853	0.124	no	0.573268
14	C9	5.167	-1.57	0.82	c2	0.080262
15	N6	-2.777	-3.057	-0.576	no	0.443075
16	C10	-1.499	-0.038	-0.545	c3	-0.100852
17	N7	-2.351	0.797	0.466	n4	0.433603
18	C11	-3.791	0.264	0.605	c3	-0.280301
19	C12	-2.343	2.234	-0.067	c3	-0.280301
20	C13	-1.728	0.756	1.834	c3	-0.211173
21	C14	-4.707	0.446	-0.602	c3	-0.561631
22	Cl1	-6.31	-0.194	-0.111	cl	-0.005086
23	C15	-3.174	3.251	0.712	c3	-0.561631
24	Cl2	-2.96	4.826	-0.124	cl	-0.005086
25	H1	3.181	1.395	-1.955	h5	0.169007
26	H2	1.973	-0.812	-1.638	h1	0.176864
27	H3	2.721	-2.042	-0.646	h1	0.176864
28	H4	1.991	-0.73	1.456	h1	0.068138
29	H5	1.16	0.404	0.409	h1	0.068138
30	H6	1.292	-3.268	1.308	h5	0.188284
31	O1	7.825	1.582	-0.56	o	-0.306725
32	H7	6.136	-1.658	1.297	ha	0.179408
33	H8	4.451	-2.369	0.977	ha	0.179408
34	O2	-3.093	-4.208	-0.431	o	-0.284316
35	O3	-3.417	-2.185	-1.174	o	-0.284316
36	H9	-0.696	0.635	-0.847	hx	0.108222
37	H10	-2.14	-0.209	-1.405	hx	0.108222
38	H11	-3.709	-0.791	0.854	hx	0.189977
39	H12	-4.213	0.777	1.468	hx	0.189977
40	H13	-1.301	2.555	-0.068	hx	0.189977
41	H14	-2.68	2.19	-1.102	hx	0.189977
42	H15	-0.716	1.156	1.783	hx	0.124069
43	H16	-2.315	1.361	2.519	hx	0.124069
44	H17	-1.715	-0.269	2.193	hx	0.124069
45	H18	-4.855	1.488	-0.876	h1	0.293485
46	H19	-4.39	-0.128	-1.467	h1	0.293485
47	H20	-4.239	3.03	0.711	h1	0.293485
48	H21	-2.833	3.387	1.736	h1	0.293485
49	O4	7.463	0.26	1.149	o	-0.306725

Species **4** (GAFF)

Atom #	Label	x	y	z	Atom type	Point charge
1	C1	5.412	0.496	-0.328	cc	0.467948
2	N1	4.839	1.19	-1.335	nd	-0.521775
3	C2	4.657	-0.652	0.053	cc	-0.130794
4	C3	3.736	0.535	-1.613	cd	0.144853
5	N2	3.556	-0.577	-0.825	na	-0.00077
6	C4	2.384	-1.425	-0.809	c3	-0.184041
7	C5	1.458	-1.056	0.376	c3	0.03652
8	N3	0.222	-1.846	0.385	na	-0.009364
9	C6	-1.043	-1.511	-0.1	cc	-0.326051
10	C7	0.181	-3.177	0.707	cc	0.135136
11	C8	-1.734	-2.718	-0.035	cd	0.526047
12	N4	-0.988	-3.725	0.465	nd	-0.507893
13	N5	6.655	0.929	0.26	no	0.563035
14	C9	4.859	-1.643	0.994	c2	-0.334104
15	N6	-3.086	-2.98	-0.504	no	0.456407
16	C10	-1.416	-0.157	-0.593	c3	-0.117764
17	N7	-2.061	0.829	0.447	n4	0.282847
18	C11	-3.554	0.534	0.667	c3	-0.258739
19	C12	-1.838	2.235	-0.116	c3	-0.258739
20	C13	-1.381	0.702	1.785	c3	-0.146539
21	C14	-4.486	0.854	-0.503	c3	-0.504876
22	Cl1	-6.155	0.515	0.071	cl	-0.026586
23	C15	-2.453	3.383	0.688	c3	-0.504876
24	Cl2	-2.064	4.9	-0.193	cl	-0.026586
25	H1	3.035	0.801	-2.395	h5	0.156242
26	H2	1.854	-1.298	-1.758	h1	0.137747
27	H3	2.685	-2.475	-0.744	h1	0.137747
28	H4	1.977	-1.218	1.324	h1	0.071171
29	H5	1.211	0.006	0.322	h1	0.071171
30	H6	1.038	-3.695	1.117	h5	0.202428
31	O1	7.178	1.942	-0.188	o	-0.381685
32	H7	5.744	-1.612	1.611	ha	0.183814
33	H8	4.177	-2.479	1.097	ha	0.183814
34	O2	-3.521	-4.109	-0.413	o	-0.308812
35	O3	-3.69	-2.005	-0.985	o	-0.308812
36	H9	-0.539	0.375	-0.965	hx	0.142769
37	H10	-2.141	-0.248	-1.399	hx	0.142769

38	H11	-3.624	-0.525	0.911	hx	0.187964
39	H12	-3.849	1.115	1.543	hx	0.187964
40	H13	-0.757	2.377	-0.173	hx	0.187964
41	H14	-2.233	2.236	-1.133	hx	0.187964
42	H15	-0.321	0.931	1.676	hx	0.118408
43	H16	-1.834	1.402	2.486	hx	0.118408
44	H17	-1.514	-0.315	2.155	hx	0.118408
45	H18	-4.459	1.902	-0.802	h1	0.272737
46	H19	-4.309	0.214	-1.365	h1	0.272737
47	H20	-3.539	3.321	0.766	h1	0.272737
48	H21	-2.025	3.474	1.688	h1	0.272737
49	O4	7.094	0.236	1.188	o	-0.381685

Species **6** (Amber force field. Capping atoms have charge = 0)

Atom #	Label	x	y	z	Atom type	Point charge
1	P1	-2.449	3.904	-0.243	P	1.13125
2	O1	-2.831	3.695	1.201	O2	-0.776
3	O2	-1	3.954	-0.635	O2	-0.776
4	O3	-3.097	2.665	-1.123	OS	-0.3617
5	C1	-4.485	2.354	-1.062	CT	-0.144
6	H1	-4.826	2.235	-2.095	H1	0.10488
7	H2	-5.061	3.163	-0.603	H1	0.10488
8	C2	-4.765	1.049	-0.324	CT	0.15824
9	H3	-5.822	0.801	-0.454	H1	0.08383
10	O4	-3.977	-0.009	-0.942	OS	-0.4544
11	C3	-3.236	-0.647	0.063	CT	0.2848
12	H4	-3.769	-1.521	0.45	H2	0.10461
13	N1	-1.988	-1.214	-0.494	N*	-0.1397
14	C4	-0.744	-0.808	-0.216	CR	0.18464
15	H5	-0.505	0.102	0.308	H5	0.25444
16	N2	0.143	-1.675	-0.697	N*	-0.1508
17	C5	-0.556	-2.676	-1.354	CB	-0.3799
18	C6	-0.111	-3.817	-2.093	C	0.80174
19	O5	1.025	-4.192	-2.323	O	-0.6176
20	N3	-1.237	-4.527	-2.581	NA	-0.7499
21	H6	-1.002	-5.322	-3.16	H	0.41585
22	C7	-2.557	-4.184	-2.371	CA	0.95323
23	N4	-3.506	-4.97	-2.928	N2	-0.9988
24	H7	-3.311	-5.913	-3.22	H	0.45332
25	H8	-4.459	-4.749	-2.686	H	0.45332
26	N5	-2.924	-3.122	-1.687	NC	-0.664
27	C8	-1.902	-2.393	-1.223	CB	0.58113

28	C9	-4.438	0.992	1.188	CT	0.51252
29	H9	-4.426	1.975	1.66	H1	0.06365
30	C10	-3.042	0.364	1.181	CT	-0.1403
31	H10	-2.351	1.163	0.909	HC	0.05001
32	H11	-2.755	-0.07	2.136	HC	0.05001
33	O6	-5.448	0.166	1.802	OS	-0.6887
34	C11	1.758	0.926	-1.827	CK	0.46917
35	N6	2.507	2.04	-1.673	NB	-0.4197
36	C12	2.123	-0.095	-0.96	CA	-0.2985
37	C13	3.356	1.741	-0.733	CK	0.02785
38	N7	3.196	0.463	-0.248	N*	0.07242
39	C14	4.182	-0.138	0.663	CT	-0.029
40	C15	5.428	-0.703	-0.092	CT	-0.1036
41	N8	6.671	-0.708	0.685	N*	-0.0206
42	C16	7.642	0.341	0.746	C*	-0.3402
43	C17	7.187	-1.735	1.415	CK	0.4219
44	C18	8.688	-0.237	1.564	CK	0.67285
45	N9	8.376	-1.477	1.945	NB	-0.5222
46	N10	0.733	0.883	-2.872	N	0.46806
47	C19	1.623	-1.505	-0.754	CT	0.08287
48	N11	9.981	0.379	1.953	N	0.50511
49	C20	7.538	1.554	0.137	CM	0.14681
50	H12	4.108	2.407	-0.335	H5	0.18477
51	H13	4.496	0.647	1.352	H1	0.09458
52	H14	3.706	-0.912	1.266	H1	0.09458
53	H15	5.243	-1.726	-0.424	H1	0.13032
54	H16	5.603	-0.11	-0.991	H1	0.13032
55	H17	6.667	-2.675	1.533	H5	0.18756
56	O7	0.539	1.878	-3.525	OS	-0.3213
57	H18	1.944	-2.159	-1.565	H1	0.12927
58	H19	2.034	-1.918	0.17	H1	0.12927
59	O8	10.171	1.526	1.547	OS	-0.2997
60	O9	10.739	-0.286	2.63	OS	-0.2997
61	H20	6.681	1.828	-0.466	HA	0.16241
62	H21	8.341	2.267	0.243	HA	0.16241
63	O10	0.176	-0.21	-3.027	OS	-0.3213
64	P2	-5.162	-0.873	3.04	P	0
65	O11	-4.526	-2.134	2.488	O2	0
66	O12	-4.406	-0.203	4.158	O2	0
67	O13	-6.718	-1.136	3.424	OS	0
68	C21	-7.018	-2.092	4.453	CT	0
69	H22	-6.579	-1.782	5.405	H1	0
70	H23	-6.641	-3.081	4.18	H1	0

71	H24	-8.103	-2.12	4.541	H1	0
72	O14	-3.292	5.198	-0.772	OS	0
73	C22	-3.079	5.776	-2.064	CT	0
74	H25	-2.046	6.115	-2.169	H1	0
75	H26	-3.301	5.058	-2.859	H1	0
76	H27	-3.758	6.625	-2.14	H1	0

Species **9** (Amber force field. Capping atoms have charge = 0)

Atom #	Label	x	y	z	Atom type	Point charge
1	P1	3.521	3.802	-0.354	P	1.2512
2	O1	4.751	3.183	-0.937	O2	-0.7705
3	O2	2.279	4.043	-1.18	O2	-0.7705
4	O3	2.965	2.815	0.878	OS	-0.3696
5	C1	3.815	1.851	1.485	CT	-0.2027
6	H1	3.645	1.896	2.565	H1	0.1402
7	H2	4.868	2.062	1.283	H1	0.1402
8	C2	3.491	0.44	1.007	CT	0.0381
9	H3	4.08	-0.28	1.579	H1	0.1196
10	O4	2.088	0.129	1.281	OS	-0.2984
11	C3	1.335	0.201	0.095	CT	-0.1237
12	H4	0.88	-0.777	-0.087	H2	0.2196
13	N1	0.192	1.134	0.318	N*	-0.0616
14	C4	0.029	2.448	-0.141	CK	0.3604
15	H5	0.785	2.957	-0.743	H5	0.1886
16	N2	-1.075	3.004	0.295	NB	-0.6329
17	C5	-1.654	2.06	1.096	CB	0.0826
18	C6	-2.853	2.104	1.828	CA	0.8434
19	N3	-3.661	3.161	1.812	N2	-1.0189
20	H6	-4.506	3.162	2.361	H	0.4739
21	H7	-3.405	3.98	1.281	H	0.4739
22	N4	-3.202	1.025	2.582	NC	-0.7427
23	C7	-2.417	-0.014	2.603	CA	0.3722
24	H8	-2.687	-0.855	3.233	H5	0.1446
25	N5	-1.254	-0.173	1.904	N*	0.0436
26	C8	-0.871	0.903	1.137	CB	0.0972
27	C9	3.638	0.154	-0.49	CT	0.2540
28	H9	4.458	0.695	-0.961	H1	0.1620
29	C10	2.269	0.561	-1.061	CT	0.1447
30	H10	2.268	1.622	-1.295	HC	0.0043
31	H11	2.036	-0.004	-1.962	HC	0.0043
32	O5	3.827	-1.267	-0.589	OS	-0.5280
33	C11	-0.292	-3.876	1.365	CK	0.5161

34	N6	-0.634	-4.691	0.34	NB	-0.4765
35	C12	-0.756	-2.581	1.203	CA	-0.4053
36	C13	-1.306	-3.928	-0.473	CK	0.0016
37	N7	-1.435	-2.633	-0.008	N*	0.0627
38	C14	-2.16	-1.577	-0.726	CT	0.3914
39	C15	-3.418	-2.119	-1.442	CT	-0.5318
40	N8	-4.37	-1.051	-1.736	N*	0.2220
41	C16	-5.333	-0.524	-0.843	C*	-0.4078
42	C17	-4.506	-0.385	-2.909	CK	0.3272
43	C18	-5.976	0.475	-1.66	CK	0.6493
44	N9	-5.466	0.533	-2.886	NB	-0.4838
45	N10	0.478	-4.382	2.493	N	0.5804
46	C19	-0.466	-1.411	2.116	CT	-0.2003
47	N11	-7.069	1.337	-1.253	N	0.5085
48	C20	-5.539	-0.94	0.436	CM	0.0916
49	H12	-1.721	-4.245	-1.417	H5	0.2306
50	H13	-2.48	-0.828	-0.011	H1	-0.0296
51	H14	-1.496	-1.092	-1.446	H1	-0.0296
52	H15	-3.165	-2.6	-2.387	H1	0.2072
53	H16	-3.919	-2.866	-0.824	H1	0.2072
54	H17	-3.887	-0.596	-3.77	H5	0.2244
55	O6	0.824	-5.525	2.485	OS	-0.3746
56	H18	0.594	-1.149	2.08	H1	0.1547
57	H19	-0.642	-1.73	3.14	H1	0.1547
58	O7	-7.57	2.058	-2.096	OS	-0.3033
59	O8	-7.398	1.271	-0.064	OS	-0.3033
60	H20	-4.968	-1.748	0.874	HA	0.1757
61	H21	-6.306	-0.468	1.029	HA	0.1757
62	O9	0.712	-3.562	3.392	OS	-0.3746
63	O10	3.948	5.147	0.45	OS	0
64	C21	2.952	5.953	1.08	CT	0
65	H22	2.444	5.393	1.872	H1	0
66	H23	2.216	6.301	0.352	H1	0
67	H24	3.471	6.806	1.516	H1	0
68	P2	4.138	-1.999	-2.038	P	0
69	O11	5.311	-1.314	-2.688	O2	0
70	O12	2.854	-2.091	-2.825	O2	0
71	O13	4.495	-3.469	-1.485	OS	0
72	C22	5.727	-3.712	-0.795	CT	0
73	H25	5.779	-4.786	-0.627	H1	0
74	H26	5.737	-3.19	0.165	H1	0
75	H27	6.577	-3.389	-1.398	H1	0
